# Monomethyl fumarate confers cardioprotection after myocardial infarction via HCAR2-dependent activation of PI3K/Akt signaling

**DOI:** 10.1038/s41420-025-02927-6

**Published:** 2025-12-30

**Authors:** Yifeng Zhang, Yu Gui, Darrell Belke, Xiaopu Wang, Wen Su, Maojun Liu, Binjie Yan, Jiaxing Sun, Xinqun Hu, Xi-Long Zheng

**Affiliations:** 1 Departments of Biochemistry & Molecular Biology and Physiology & Pharmacology, Cumming School of Medicine, University of Calgary, Calgary, AB Canada; 2 Department of Cardiovascular Medicine, The Affiliated Changsha Central Hospital, Hengyang Medical School, University of South China, Changsha, China; 3 Department of Cardiology, The Second Xiangya Hospital of Central South University, Changsha, China

**Keywords:** Myocardial infarction, Drug development

## Abstract

Monomethyl fumarate (MMF), the active metabolite of dimethyl fumarate, an immunomodulatory drug approved for multiple sclerosis and psoriasis, has emerging potential in ischemic heart disease. We investigated whether MMF can attenuate myocardial infarction (MI) injury and delineated the underlying mechanisms, focusing on hydroxycarboxylic acid receptor 2 (HCAR2, also known as GPR109A) and PI3K/Akt signaling. In a mouse MI model induced by permanent left anterior descending coronary artery ligation, MMF administration prior to ischemia significantly preserved left ventricular function and reduced cardiomyocyte apoptosis compared with untreated MI. Echocardiography and pressure–volume loop analyses demonstrated higher ejection fraction and cardiac output in MMF-treated MI mice, accompanied by attenuation of adverse ventricular remodeling. TUNEL staining and analysis of apoptotic markers showed that MMF decreased myocardial cell death and caspase-3 activation in vivo, while concomitantly upregulating HCAR2 expression and enhancing Akt phosphorylation in ischemic myocardium. In vitro, MMF protected HL-1 cardiomyocytes from CoCl₂-induced hypoxic injury, improving cell viability and reducing apoptosis, as evidenced by fewer TUNEL-positive cells and a lower Bax/Bcl-2 ratio compared with hypoxia alone. Pharmacological inhibition of Gi-coupled signaling with pertussis toxin or siRNA-mediated knockdown of HCAR2 abolished MMF’s cytoprotective effects and blunted MMF-induced Akt phosphorylation, and PI3K/Akt pathway inhibition eliminated MMF’s anti-apoptotic benefits in vitro. Collectively, these findings demonstrate that MMF markedly reduces ischemic cardiomyocyte injury via an HCAR2-dependent mechanism involving activation of the pro-survival PI3K/Akt pathway, establishing a novel cardioprotective role for MMF and supporting its translational potential as a therapeutic strategy to mitigate acute MI injury.

## Introduction

Ischemic heart disease, particularly acute myocardial infarction (MI), remains a leading cause of morbidity and mortality worldwide. Reperfusion strategies (primary percutaneous coronary intervention or thrombolysis) are the cornerstone of acute MI treatment and have dramatically improved outcomes. However, reperfusion also triggers additional injury to jeopardized myocardium (ischemia-reperfusion injury), and the loss of cardiomyocytes from ischemic and reperfusion stress can lead to adverse remodeling and heart failure [1]. Standard post-MI pharmacotherapies—including β-blockers, ACE inhibitors, and statins—mitigate chronic remodeling and reinfarction risk [1], but there is no established therapy to directly protect the heart from acute ischemia-reperfusion injury. This gap has prompted exploration of novel cardioprotective agents, particularly those targeting inflammation and oxidative stress, which are key contributors to myocardial injury [1].

Dimethyl fumarate (DMF) is an oral immunomodulatory drug approved for relapsing multiple sclerosis and psoriasis. After ingestion, DMF is rapidly hydrolyzed to MMF, which is the primary active metabolite [2]. DMF/MMF have well-documented anti-oxidative and anti-inflammatory effects, principally via activation of the NRF2 (nuclear factor erythroid 2–related factor 2) pathway and inhibition of NF-κB signaling [1]. In preclinical studies, DMF consistently reduced infarct size and improved cardiac function in rodent models of MI [2]. For example, treatment with DMF (10 mg/kg) in rats prior to coronary occlusion limited infarct size, an effect linked to suppression of NF-κB–mediated inflammation [2]. In a recent study, post-MI administration of DMF in mice attenuated adverse left ventricular remodeling, enhanced angiogenesis, and augmented markers of antioxidative defense (e.g., Nrf2), further supporting the cardioprotective potential of fumarate derivatives [2]. Notably, NRF2 activation has been identified as a major mechanism for the protective effects of DMF in cardiac I/R injury models [2, 3]. At the same time, emerging evidence suggests that DMF/MMF may exert protective effects via additional pathways and targets beyond Nrf2 [4].

One such target is the hydroxycarboxylic acid receptor 2 (HCAR2), also known as GPR109A or the niacin receptor. HCAR2 is a Gαi protein-coupled receptor expressed on various cell types including immune cells (monocytes/macrophages, neutrophils) and adipocytes [4]. MMF is a known agonist of HCAR2 [5], and HCAR2 activation has been implicated in the immunomodulatory actions of fumarates. In experimental autoimmune encephalomyelitis (a multiple sclerosis model), the therapeutic effect of DMF was abrogated in HCAR2-deficient mice, indicating HCAR2 is required for MMF/DMF efficacy in that context [6]. Cellular studies have shown that MMF binding to HCAR2 can switch immune cells from a pro-inflammatory to an anti-inflammatory phenotype via downstream signaling cascades [4]. For instance, in microglia, HCAR2 activation by MMF engaged an AMPK–Sirt1 pathway that ultimately inhibited NF-κB and reduced pro-inflammatory cytokine production [4]. These findings underscore that HCAR2 is a pivotal receptor mediating some of the anti-inflammatory and cytoprotective effects of MMF in the immune and central nervous systems. However, the role of HCAR2 in the heart, especially in the setting of acute MI, is not well characterized. Given that sterile inflammation and immune cell infiltration are critical in acute MI injury and subsequent remodeling, it is pertinent to investigate if activating HCAR2 could confer cardioprotection by modulating these processes.

Activation of cell-survival signaling pathways is another hallmark of cardioprotective interventions (such as ischemic preconditioning). Among these, the PI3K/Akt pathway is particularly well known to promote cardiomyocyte survival and limit apoptosis during ischemic stress [1, 3]. Akt (protein kinase B) activation can inhibit pro-apoptotic factors (e.g., Bax, caspase-9) and enhance pro-survival factors (e.g., Bcl-2), thereby reducing cell death in the ischemic heart. Previous studies with DMF have hinted at Akt involvement; for example, in vitro DMF treatment of cardiomyoblasts under simulated I/R increased p-Akt levels in parallel with Nrf2 activation [1]. Whether MMF can directly activate Akt signaling in cardiomyocytes via a receptor-mediated mechanism is an intriguing question. HCAR2 signals through Gαi proteins, which classically reduce cAMP, but βγ subunits of Gαi can also engage PI3K, potentially linking HCAR2 stimulation to Akt activation. We hypothesized that MMF may activate HCAR2 in cardiac cells, leading to PI3K/Akt pathway activation and resultant anti-apoptotic, cardioprotective effects.

In this study, we evaluated the therapeutic potential of MMF in a mouse model of acute MI and in cultured cardiomyocytes under hypoxic stress. We specifically examined the role of HCAR2 and the PI3K/Akt signaling pathway in mediating MMF’s effects. By using pharmacological inhibitors and gene knockdown approaches, we aimed to clarify the mechanistic basis of MMF-induced cardioprotection. Our findings demonstrate that MMF markedly attenuates myocardial injury after infarction, via HCAR2-dependent activation of PI3K/Akt signaling, highlighting a novel mechanism of cardioprotection and a promising avenue for translational therapy.

## Results

### MMF reduces infarct size, early mortality, and ventricular remodeling post-MI

**Fig. 1:**
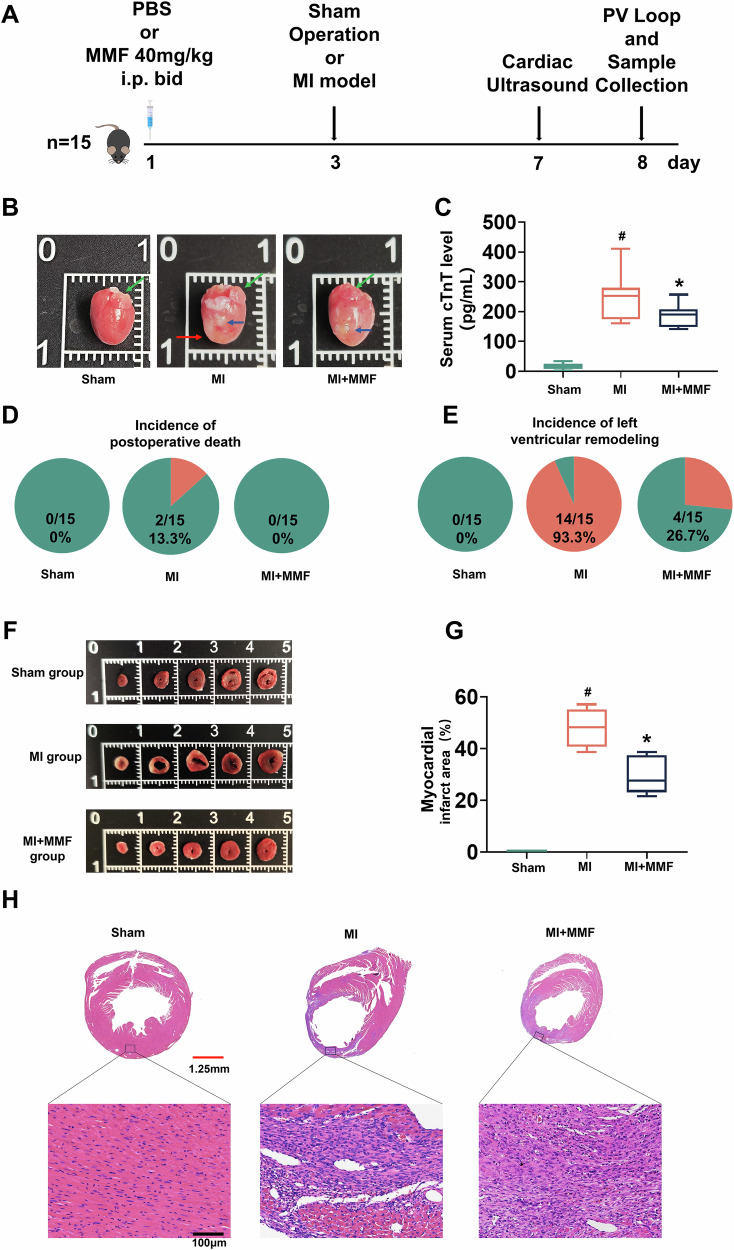
MMF treatment reduces acute infarct size, mortality, and cardiac remodeling after MI. **A** Experimental timeline for MI model and MMF treatment. Mice (*n* = 15 per group) received PBS or MMF (40 mg/kg i.p. BID) starting 2 days prior to surgery. On day 3, mice underwent either sham operation or myocardial infarction (MI) by coronary occlusion. Cardiac ultrasound was performed at day 7, and invasive pressure-volume (PV) analysis and tissue collection at day 8 post-MI. **B** Representative gross anatomy of hearts on day 8 from Sham, MI, and MI + MMF groups. Green arrows indicate the left atrial appendage, serving as an anatomical landmark. The blue arrows indicate the ligation site of the LAD coronary artery. The red arrows highlight the morphological changes in ventricular remodeling. **C** Serum cardiac troponin T (cTnT) levels at 7 days post-MI (box-and-whisker plots). cTnT, a marker of cardiomyocyte injury, is markedly elevated in MI mice compared to Sham, and significantly reduced in the MMF-treated group (*n* = 10 per group, #*p* < 0.05 vs Sham; *p < 0.05 vs MI). **D** Incidence of postoperative death by day 8 in each group (pie charts). Two of 15 MI mice died (13.3% mortality, red segment), whereas no deaths occurred in Sham or MI + MMF groups (0/15). **E** Incidence of adverse left ventricular (LV) remodeling in each group, based on echocardiographic criteria (pie charts). Among all surgically induced MI animals (*n* = 15), 14 (including two non-survivors with confirmed cardiac rupture) developed dilated, dysfunctional LV remodeling (red segment), versus 0% in Sham and only 26.7% (4 of 15) in MMF-treated mice—indicating that MMF greatly prevented pathological remodeling. **F** Representative cross-sections of the heart (five 1 mm slices, apex to base) stained to delineate infarct area (pale) versus viable myocardium (red). Sham hearts show no infarction; MI hearts exhibit a large pale infarct across multiple slices; MI + MMF hearts have substantially smaller infarcted regions. **G** Quantification of myocardial infarct size expressed as percentage of LV volume (box plots for each group). Infarct size is significantly smaller in MMF-treated mice compared to untreated MI (*n* = 10 per group, *p < 0.05 vs MI). **H** Histological sections of the LV (H&E staining) on day 8. Top: low-magnification views of entire LV cross-sections (scale bar = 2 mm); bottom: high-magnification views of the infarct border zone (scale bar = 50 µm). Sham myocardium shows normal cardiac muscle structure. MI myocardium shows extensive coagulative necrosis, inflammatory cell infiltration, and wall thinning in the infarct region. MI + MMF hearts exhibit preserved myocardial fibers and reduced inflammatory damage in the injured area. *Statistical symbols: # p* < 0.05 vs Sham; *p < 0.05 vs MI *(*one-way ANOVA). MMF monomethyl fumarate, MI myocardial infarction, PBS phosphate‑buffered saline, cTnT cardiac troponin T, PV loop, pressure–volume loop, i.p. intraperitoneal injection, BID, twice daily.

To investigate the effect of MMF, we have pretreated mice for two days before perfoming the perment ligaation of the MI mouse model. The timeline has been outlined in Fig. 1A. In brief, mice received either vehicle (PBS) or MMF (40 mg/kg i.p., twice daily) starting 2 days before MI surgery (or sham operation), with cardiac assessments at day 5 post-MI before sarcrificing animals for tissue collection. MMF had a striking protective effect on acute MI outcomes. Gross examination of hearts at day 5 (Fig. 1B) revealed pronounced ventricular remodeling in MI hearts, characterized by dilated chambers and thin ventricular walls (red arrows), whereas MMF-treated hearts maintained near-normal cardiac architecture with minimal remodeling, grossly resembling sham controls (Fig. 1B). Consistently, serum cardiac troponin T (cTnT)—a biomarker of myocardial injury that correlates with infarct size [7]—was markedly elevated in MI mice (~400 pg/mL) but was significantly reduced with MMF treatment (Fig. 1C). MMF-treated mice also had improved survival: 2 of 15 mice (13.3%) in the untreated MI group died post-surgery, whereas no deaths occurred in either sham or MMF-treated groups by day 8 (Fig. 1D). Furthermore, MMF attenuated adverse cardiac remodeling. By 1 week, 93.3% of surviving MI mice showed signs of left ventricular (LV) dilatation and dysfunction (pie chart in Fig. 1E), reflecting the high incidence of post-MI ventricular remodeling. In contrast, only 26.7% of MMF-treated MI mice exhibited significant LV remodeling (comparable to 0% in shams), indicating that MMF largely prevented pathological remodeling of the LV. Histological and morphometric analyses confirmed a reduction in infarct size with MMF. Figure 1F shows representative transverse LV slices (apex to base) stained to delineate infarcted myocardium; untreated MI hearts displayed extensive infarcted areas (pale regions) across multiple slices, whereas MMF-treated hearts had much smaller infarct regions. Quantification revealed that MI resulted in ~50% of the LV being infarcted, while MMF treatment reduced infarct size to ~30% of the LV (Fig. 1G, *p* < 0.05 vs MI). Correspondingly, hematoxylin and eosin (H&E) staining of myocardial sections (Fig. 1H) demonstrated massive cardiomyocyte loss with inflammatory cell infiltration in the infarct zone of untreated MI hearts, whereas MMF-treated hearts showed more preserved myocardium and less inflammation in the injured area. Together, these results show that MMF significantly limited the acute damage from MI—reducing cardiomyocyte injury and death, improving early survival, and mitigating LV remodeling.

### MMF preserves cardiac function after MI

**Fig. 2:**
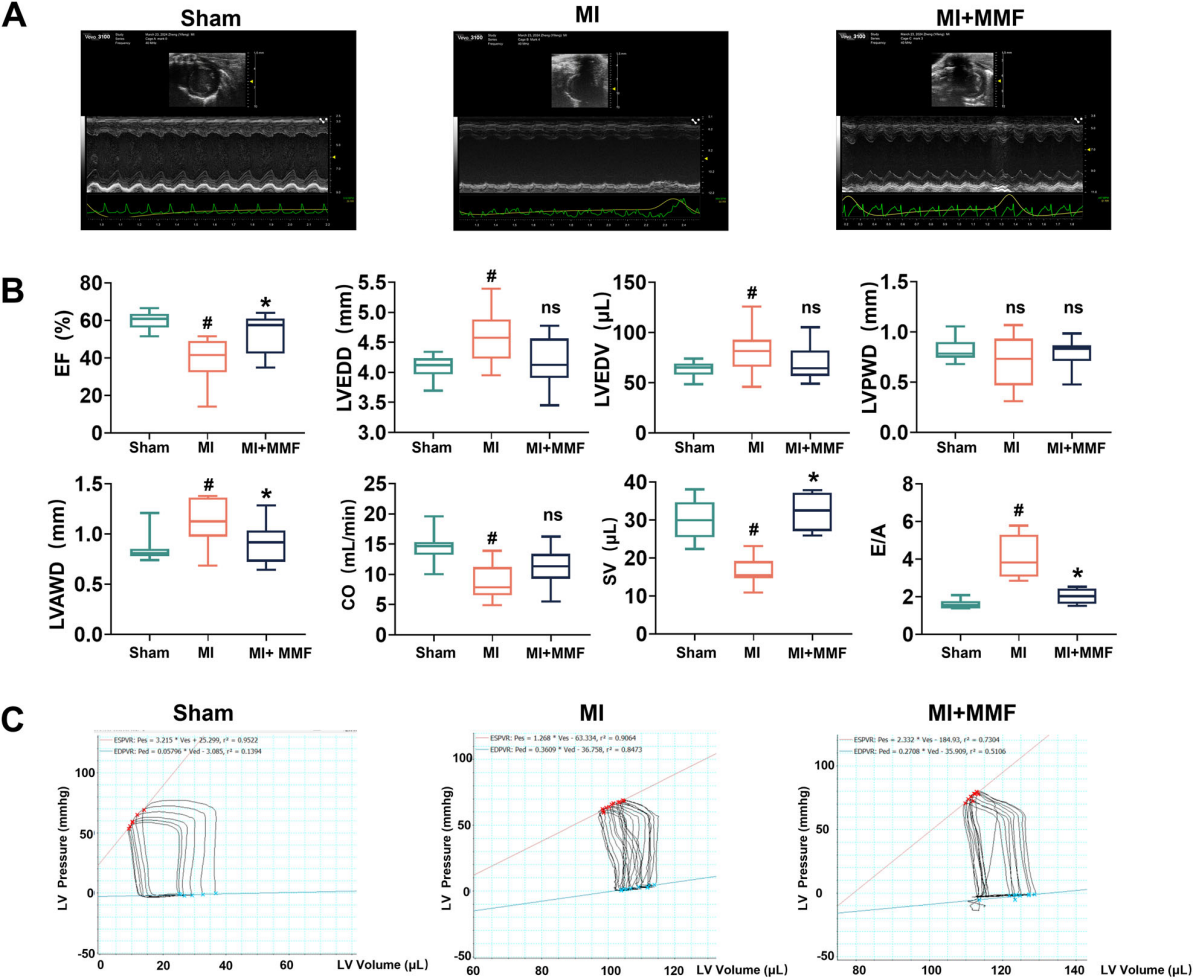
Echocardiographic and hemodynamic assessments showing preserved cardiac function with MMF after MI. **A** Representative M-mode echocardiography images of the left ventricle in a Sham mouse, an untreated MI mouse, and an MI + MMF mouse at 7 days post-MI. The MI heart shows reduced wall motion and chamber dilation, whereas the MMF-treated heart has enhanced contractility and smaller chamber dimensions relative to MI. **B** Quantitative echocardiographic parameters (box-and-whisker plots) measured at 1 week post-MI: Ejection fraction (EF, %), LV end-diastolic diameter (LVEDD, mm), LV end-diastolic volume (LVEDV, µL), LV posterior wall thickness in diastole (LVPWT, mm), LV anterior wall thickness in diastole (LVAWd, mm), cardiac output (CO, mL/min), stroke volume (SV, µL), and the early-to-late transmitral flow ratio (E/A). MI causes a significant decline in EF, CO, and SV, an increase in LVEDD and LVEDV (ventricular dilation), and thinning of the anterior wall (lower LVAWd), indicating systolic dysfunction and adverse remodeling. MMF treatment partially preserves EF and SV and prevents wall thinning and extreme dilation (e.g., higher LVAWd and smaller LVEDV vs MI). Diastolic function (E/A ratio) is also improved by MMF. #*p* < 0.05 vs Sham; *p < 0.05 vs MI. **C** Representative left ventricular pressure-volume (PV) loops obtained from terminal PV analysis in each group on day 8. The Sham heart exhibits a large, robust PV loop (normal contractility and volume). The MI heart’s PV loop is right-shifted with reduced peak pressure and stroke volume (smaller loop area), reflecting decreased contractile function and increased end-diastolic volume. The MMF-treated heart shows an improved PV loop compared to MI—higher systolic pressure and larger loop area—indicating better preserved contractile function and less ventricular dilation. These data demonstrate that MMF therapy mitigates the loss of cardiac function after MI. MMF monomethyl fumarate, MI myocardial infarction, PV loop, pressure–volume loop, EF ejection fraction, CO cardiac output, SV stroke volume, E/A E-wave to A-wave ratio, LVAWD left ventricular anterior wall diastole, LVPWD left ventricular Posterior wall diastole, LVEDD left ventricular end diastolic diameter, LVEDV left ventricular end diastolic volume.

Echocardiography and hemodynamic measurements indicated that MMF preserved cardiac function post-MI. Figure 2A shows representative M-mode echocardiograms from each group post‑MI day 5. MI caused a clear decline in systolic function, evidenced by reduced wall motion in the MI heart, whereas the MMF-treated heart retained more vigorous contractions similar to sham. Quantitative echocardiographic parameters are summarized in Fig. 2B. MI induced a significant drop in ejection fraction (EF) (to ~40% in MI vs ~65% in sham, *p* < 0.05), reflecting systolic dysfunction. MMF treatment partially rescued systolic function, with EF ~ 50% (*p* < 0.05 vs MI) in treated mice. End-diastolic dimensions and volumes were increased by MI (e.g., LV end-diastolic diameter (LVEDD) and volume (LVEDV) were significantly elevated in MI vs sham, indicating ventricular dilatation), while MMF tended to blunt these increases (LVEDD and LVEDV in MMF group were intermediate between sham and MI). Notably, MI caused thinning of the infarcted ventricular wall: LV anterior wall thickness in diastole (LVAWd) was reduced in MI vs sham (*p* < 0.05), whereas MMF preserved anterior wall thickness (significantly higher than MI, Fig. 2B). Stroke volume (SV) and cardiac output (CO) were also depressed by MI (SV fell by ~25%, CO by ~30% vs sham), consistent with impaired pump function. MMF-treated mice showed higher SV and CO than untreated MI—for instance, SV in MMF mice was restored toward normal (∼30 µL vs ~27 µL in MI, *p* < 0.05 vs MI). In addition, diastolic function was improved by MMF: MI mice exhibited an altered transmitral flow ratio (E/A ratio ~1.2 vs ~1.8 in sham, suggesting impaired relaxation), whereas MMF treatment maintained a higher E/A ratio (~1.6, *p* < 0.05 vs MI). Table 1 presents detailed hemodynamic parameters obtained from LV pressure-volume (PV) loop analysis at day 8, which corroborate the echocardiographic findings. Briefly, MI induced significant systolic dysfunction and stiffening: maximal LV pressure (Pmax) and dP/dt_max (contractility index) were both reduced in MI vs sham, while end-systolic volume (ESV) and end-diastolic pressure (EDP) were markedly increased (all *p* < 0.05). MMF treatment improved these indices—for example, Pmax and dP/dt_max in MMF mice were significantly higher than in MI mice (approaching sham values), and EDP was normalized (3.9 mmHg in MMF vs 12.6 mmHg in MI, *p* < 0.05). MMF also prevented the rise in the LV relaxation time constant (Tau) seen in MI, indicating better diastolic relaxation (Tau remained ~9 ms in MMF vs 16 ms in MI). Other PV-loop measures of ventricular stiffness and efficiency showed favorable shifts with MMF (Table 1): the end-systolic PV relationship (ESPVR) slope moved toward sham values in the MI + MMF group relative to MI, and stroke work (SW) was partially restored with MMF. Representative PV loops from each group (Fig. 2C) illustrate these differences in cardiac performance: the MI loop is rightward-shifted with a smaller area (reduced stroke work) and lower peak pressure, whereas the MMF-treated heart’s PV loop is notably improved—exhibiting higher developed pressure and larger loop area—compared to untreated MI. Taken together, these data demonstrate that MMF significantly preserved cardiac function after MI, limiting the extent of systolic dysfunction and adverse ventricular remodeling.

**Table 1:** Hemodynamic parameters from LV pressure-volume (PV) loop analysis in Sham, MI, and MI + MMF groups (Mean ± SD, *n* = 8 mice in MI group, *n* = 10 mice in Sham and MI + MMF).

Parameter	Sham (Mean ± SD)	MI (Mean ± SD)	MI + MMF (Mean ± SD)
Heart Rate (bpm)	429.54 ± 15.78	398.28 ± 31.09*	423.42 ± 29.11#
CO (µL/min)	15706.0 ± 2530.4	11011.6 ± 2942.9*	13739.2 ± 3746.0#
SW (mmHg·µL)	2204.20 ± 288.84	1141.08 ± 513.29*	1741.16 ± 664.72#
SV (µL)	36.77 ± 6.56	27.63 ± 5.92*	32.58 ± 8.46#
Vmax (µL)	160.27 ± 50.68	107.33 ± 45.63*	167.10 ± 35.51#
Vmin (µL)	114.39 ± 42.69	107.33 ± 35.63	125.93 ± 42.75
Pmax (mmHg)	84.0 ± 6.53	72.37 ± 4.53*	81.48 ± 9.54#
Pmin (mmHg)	–2.13 ± 0.84	0.77 ± 1.09*	–0.85 ± 0.95#
dP/dt_max (mmHg/s)	6749.0 ± 1946.4	4368.02 ± 1723.30*	6231.80 ± 1260.15#
dP/dt_min (mmHg/s)	–5245.4 ± 1081.5	–2636.60 ± 711.34*	–4350.40 ± 1317.47#
EDP (mmHg)	3.07 ± 3.38	12.64 ± 6.72*	3.92 ± 5.12#
ESP (mmHg)	74.74 ± 6.64	67.32 ± 3.42*	75.38 ± 9.12#
EDV (µL)	151.37 ± 47.17	161.00 ± 34.44	145.00 ± 33.57
ESV (µL)	118.79 ± 15.20	136.81 ± 6.72*	120.25 ± 34.42#
Tau (ms)	8.57 ± 5.77	16.38 ± 6.56*	9.29 ± 3.94#
ESPVR (slope) (mmHg/µL)	3.52 ± 0.47	1.51 ± 0.42*	2.91 ± 0.18#
EDPVR (slope) (mmHg/µL)	0.18 ± 0.07	0.39 ± 0.09*	0.27 ± 0.10#
EA (mmHg/µL)	2.28 ± 0.61	2.77 ± 0.35*	2.77 ± 0.85

Mice underwent PV loop catheterization the terminal endpoint (day 5 post‑MI) to assess cardiac function. Listed are heart rate (HR, beats/min), cardiac output (CO, µL/min), stroke work (SW, mmHg·µL), stroke volume (SV, µL), maximal and minimal LV volumes (Vmax, Vmin, in µL), maximal and minimal LV pressures (Pmax, Pmin, in mmHg), maximal and minimal first derivatives of LV pressure (dP/dt_max and dP/dt_min, in mmHg/s), end-diastolic pressure (EDP, mmHg), end-systolic pressure (ESP, mmHg), end-diastolic volume (EDV, µL), end-systolic volume (ESV, µL), the time constant of LV relaxation (Tau, ms), measures of LV diastolic stiffness (EDPVR slope [mmHg/µL]), measures of LV systolic elastance (ESPVR slope [mmHg/µL]) and arterial elastance (EA, mmHg/µL). Sham mice have normal hemodynamic values. MI mice show significant systolic dysfunction and impaired diastolic properties: for example, CO, SW, and SV are all reduced vs Sham; Pmax and dP/dt_max are lower (indicative of decreased contractility); EDP and Tau are elevated (impaired relaxation); and ESV is higher (ventricular dilation). MI + MMF mice show improved metrics compared to MI—partial restoration of CO, SW, and SV, higher Pmax and dP/dt_max, lower EDP and Tau, and reduced ESV—reflecting better preserved systolic function and less diastolic dysfunction. *Symbols:* * indicates *p* < 0.05 vs Sham; # indicates *p* < 0.05 vs MI (one-way ANOVA with post hoc test).

### MMF attenuates myocardial apoptosis in vivo

**Fig. 3:**
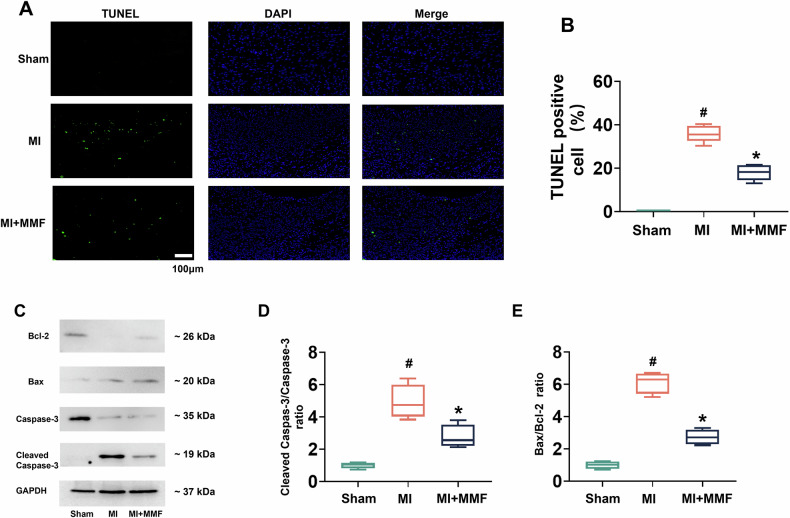
MMF suppresses cardiomyocyte apoptosis in the infarcted heart. **A** Representative fluorescence micrographs of TUNEL staining on LV tissue sections from Sham, MI, and MI + MMF groups (day 8). Apoptotic nuclei appear green (TUNEL-positive) and all nuclei are counterstained with DAPI (blue). Sham hearts show no TUNEL-positive cells. MI hearts display numerous TUNEL-positive nuclei (green) in the myocardium, indicating extensive apoptosis. MMF-treated hearts have far fewer TUNEL-positive nuclei, suggesting reduced apoptosis. Scale bar = 100 µm. **B** TUNEL-positive cells (% of total nuclei) quantified for each group (box plot). MI induces a high level of apoptosis (~40% TUNEL-positive cells vs ~0% in Sham), whereas MMF treatment significantly lowers the apoptotic fraction to ~20% (*p < 0.05 vs MI). **C** Western blots of apoptosis-related proteins in myocardial tissue (representative samples per group). Pro-apoptotic Bax is upregulated in MI and anti-apoptotic Bcl-2 is downregulated, along with increased cleaved (active) caspase-3 in MI compared to Sham. MMF treatment reverses these trends—Bax is lower, Bcl-2 higher, and cleaved caspase-3 is markedly reduced in MI + MMF hearts relative to MI. GAPDH serves as a loading control. **D**, **E** Quantification of apoptosis protein ratios from Western blots. (**D**) Cleaved caspase-3/total caspase-3 ratio, and **E** Bax/Bcl-2 ratio for each group (box plots). Both ratios are greatly elevated in MI vs Sham, consistent with increased apoptosis signaling, and both are significantly reduced in the MMF group (*p < 0.05 vs MI). These results indicate that MMF inhibits the intrinsic apoptotic pathway in the heart post-MI. *n* = 5 #*p* < 0.05 vs Sham; *p < 0.05 vs MI. MMF monomethyl fumarate, MI myocardial infarction, TUNEL terminal deoxynucleotidyl transferase dUTP Nick-End Labeling, DAPI 4’,6-diamidino-2-phenylindole, Bax Bcl-2–associated X protein, GAPDH glyceraldehyde-3-phosphate dehydrogenase.

To investigate how MMF reduced infarct injury, we examined cardiomyocyte apoptosis in the myocardium. Figure 3A shows TUNEL staining of LV sections from each group at 5 days post-MI (green fluorescence indicates apoptotic nuclei, blue = DAPI nuclear counterstain). Virtually no apoptosis was detectable in sham hearts (no TUNEL-positive nuclei). In striking contrast, MI hearts exhibited widespread apoptosis in the infarct border zone, evident as numerous bright green nuclei. Importantly, MMF treatment markedly reduced apoptosis in the post-MI heart—MMF-treated sections showed far fewer TUNEL-positive nuclei than untreated MI. Quantification of TUNEL-positive cell percentage (Fig. 3B) revealed that apoptosis levels in MI hearts reached ~40% of cells, whereas MMF-treated hearts had only ~20% TUNEL-positive cells (*p* < 0.05 vs MI). This ~50% reduction of apoptotic cell death with MMF correlates with the smaller infarcts and improved function described above, underscoring that MMF’s cardioprotection involves inhibiting cell death. At the molecular level, MMF favorably modulated the balance of pro- and anti-apoptotic proteins. Figure 3C presents Western blots of key apoptosis regulators in myocardial tissue. In MI hearts, the pro-apoptotic protein Bax was upregulated and the anti-apoptotic protein Bcl-2 was downregulated relative to sham, consistent with activation of the intrinsic apoptosis pathway after infarction [8]. Cleaved (active) caspase-3 was also greatly increased in MI, indicating executioner caspase activation. MMF reversed these changes: Bax levels were lower and Bcl-2 levels higher in MMF-treated hearts compared to MI, and cleaved caspase-3 was markedly reduced. Quantification confirmed that MI caused a high Bax/Bcl-2 ratio and high cleaved caspase-3-to-total caspase-3 ratio, while MMF brought both metrics back toward sham levels (Fig. 3D, E). For instance, the Bax/Bcl-2 ratio was elevated ~7 fold in MI vs sham, but in MMF-treated hearts this ratio was less than half of the MI value (*p* < 0.05 vs MI). Similarly, the cleaved caspase-3 fraction was several-fold higher in MI than sham, and significantly blunted by MMF (*p* < 0.05 vs MI). These data indicate that MMF inhibited myocardial apoptosis after MI, likely contributing to the preserved viable myocardium and improved outcomes. Notably, apoptosis is a reversible form of cell death that directly contributes to post-MI cardiomyocyte loss and dysfunction [8]. By reducing apoptotic cell death, MMF may help “save” endangered cardiomyocytes in the infarct region, thereby limiting infarct expansion and adverse remodeling.

### MI-induced downregulation of HCAR2 and Akt signaling is reversed by MMF

**Fig. 4:**
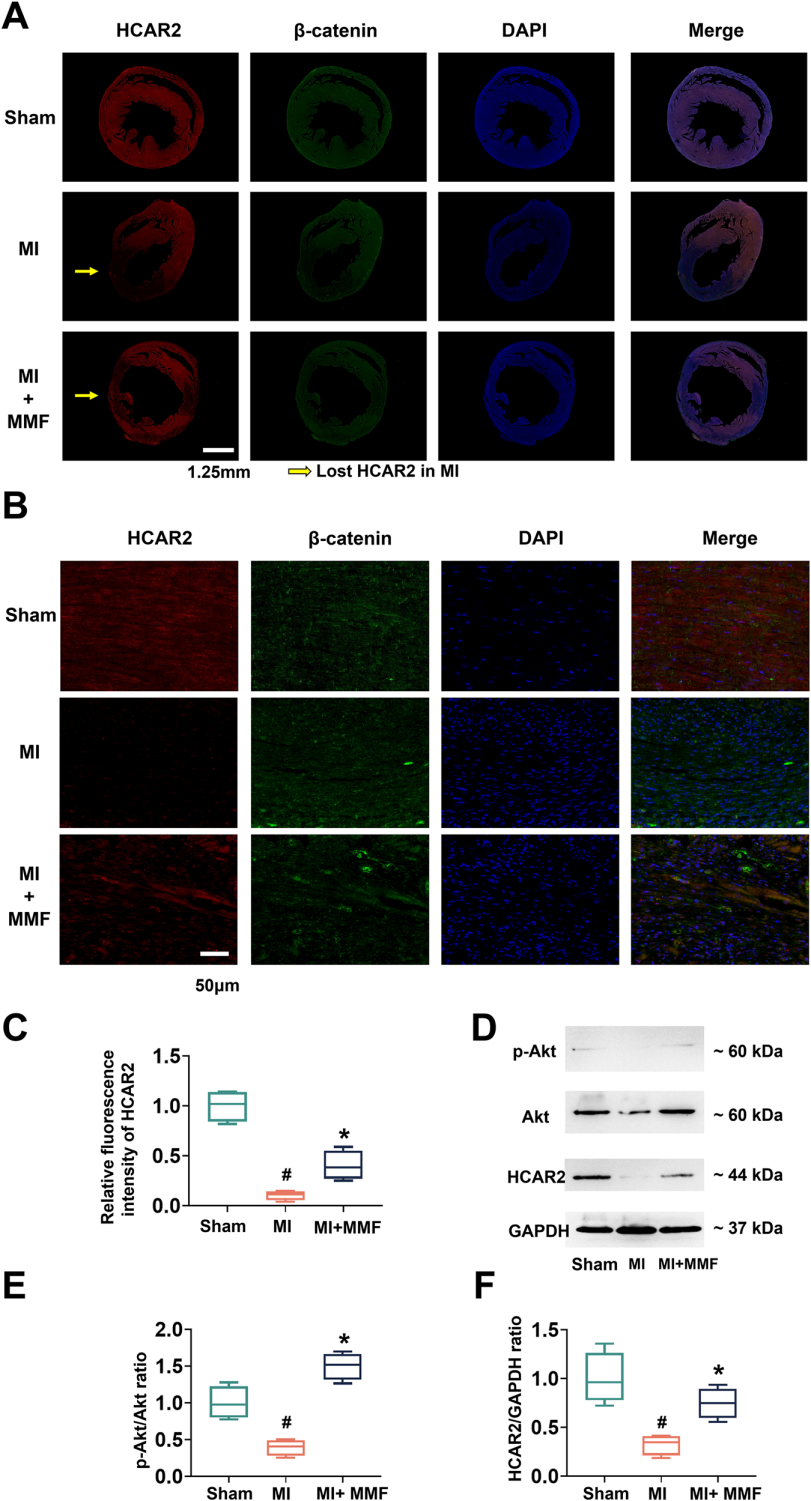
MI downregulates HCAR2 (GPR109A) and Akt activation in the heart, whereas MMF preserves HCAR2 and Akt signaling. **A** Immunofluorescence staining of transverse LV sections for HCAR2 (red) and β-catenin (green) with DAPI nuclear stain (blue), shown individually and merged. Sham hearts show strong HCAR2 expression throughout the myocardium. MI hearts have markedly diminished HCAR2 signal (especially in the infarct zone; yellow arrows indicate areas of lost HCAR2 in MI). MMF-treated MI hearts retain higher HCAR2 expression (red fluorescence is partially preserved in the LV wall). β-catenin staining (green) labels cell-cell junctions and was used as a counterstain; nuclei are blue. Scale bar = 2 mm (whole section). **B** High-magnification images of myocardium from the same groups stained for HCAR2 (red), β-catenin (green), and DAPI (blue). These panels highlight HCAR2 at the cellular level. In sham myocardium, HCAR2 is readily detected in cardiomyocytes (red); in MI myocardium, HCAR2 is nearly absent; with MMF, HCAR2 signal is visibly greater than MI alone. Scale bar = 50 µm. **C** Mean fluorescence intensity of HCAR2 immunostaining in myocardium (arbitrary units). MI significantly reduces HCAR2 intensity vs Sham (#*p* < 0.05, *n* = 4), while MMF treatment results in a higher HCAR2 signal compared to MI (*p < 0.05, *n* = 4). **D** Western blots of key proteins in LV tissue: phosphorylated Akt (p-Akt, Ser473), total Akt, HCAR2, and GAPDH. Each lane represents a different animal per group. MI causes a loss of HCAR2 protein and a major decrease in p-Akt levels relative to sham. MMF-treated hearts show increased p-Akt and higher HCAR2 protein compared to MI. **E**, **F** Quantification of Western blot data (band intensity ratios, Sham set to 1.0). **E** p-Akt/Akt ratio in each group. Akt phosphorylation is greatly diminished after MI (#*p* < 0.05 vs Sham, *n* = 4), but significantly elevated in the MMF group (*p < 0.05 vs MI, *n* = 4), indicating restored Akt activity. **F** HCAR2/GAPDH protein ratio. MI hearts exhibit dramatically lower HCAR2 levels than Sham (#*p* < 0.05, *n* = 4), whereas MI + MMF hearts have significantly higher HCAR2 expression than MI (* *p* < 0.05, *n* = 4). These data suggest that HCAR2 and downstream Akt signaling are suppressed by MI injury and that MMF prevents this suppression, potentially linking HCAR2/Akt to MMF’s protective effects. *Symbols:* #*p* < 0.05 vs Sham; *p < 0.05 vs MI. MMF monomethyl fumarate, MI myocardial infarction, DAPI 4’,6-diamidino-2-phenylindole, HCAR2 Hydroxycarboxylic Acid Receptor 2, GAPDH glyceraldehyde-3-phosphate dehydrogenase.

We next explored the molecular mechanisms underlying MMF’s cardioprotective effects, focusing on the G-protein-coupled receptor HCAR2 (GPR109A) and the pro-survival kinase Akt. HCAR2 is the high-affinity receptor for nicotinic acid (niacin), known to trigger anti-inflammatory signaling in various cells [9], but its role in the heart is not well characterized. Figure 4A reveals that myocardial HCAR2 expression is dramatically affected by MI and MMF. Heart sections were co-immunostained for HCAR2 (red) and β-catenin (green) with DAPI nuclear stain (blue). In sham hearts, HCAR2 was abundantly expressed throughout the myocardium (diffuse red signal). In contrast, MI hearts showed a profound loss of HCAR2 signal, especially in the infarct region (yellow arrows in Fig. 4A, middle row), indicating that myocardial ischemia greatly downregulates HCAR2 expression. Strikingly, MMF treatment preserved HCAR2 levels: the MMF-treated MI hearts (bottom row) maintained a much stronger HCAR2 signal than untreated MI, particularly in peri-infarct areas (arrow). Higher-magnification views (Fig. 4B) further illustrate HCAR2 localization (red) in cardiac tissue. In sham, robust HCAR2 staining is visible in cardiac muscle fibers, whereas in MI samples HCAR2 is nearly absent. With MMF, HCAR2 expression is partially retained in the myocardium. Quantification of HCAR2 fluorescence intensity (Fig. 4C) confirmed a significant reduction of HCAR2 in MI vs sham (*p* < 0.05), and a significant increase in the MMF group compared to MI (# *p* < 0.05). Parallel changes were observed in the activity of the Akt signaling pathway. Figure 4D shows Western blots of phosphorylated Akt (p-Akt), total Akt, and HCAR2 protein levels in heart tissue. Consistent with depressed HCAR2, MI hearts exhibited much lower Akt activation than shams: p-Akt (Ser473) was greatly reduced in MI (even though total Akt protein was similar), whereas MMF-treated hearts showed higher p-Akt levels. Densitometry analysis (Fig. 4E) revealed that the p-Akt/Akt ratio fell by ~70% in MI vs sham (*p* < 0.05), but in MMF-treated hearts p-Akt/Akt was restored to near-sham values (# *p* < 0.05 vs MI). Likewise, Western quantification of HCAR2 protein (Fig. 4F) showed a dramatic drop in MI (to ~25% of sham levels, *p* < 0.05) and a significant recovery with MMF (HCAR2 in MMF hearts was ~60% of sham, # *p* < 0.05 vs MI). Taken together, Fig. 4 demonstrates that myocardial infarction leads to loss of HCAR2 expression and Akt activity in the heart, and that MMF treatment counteracts these changes. Given that HCAR2 activation can trigger Gi protein signaling to activate PI3K/Akt pathways [9, 10], these findings raised the possibility that MMF might exert its anti-apoptotic effect by engaging HCAR2 and subsequently the Akt survival pathway.

### MMF protects HL-1 cardiomyocytes from hypoxia-induced apoptosis in vitro

**Fig. 5:**
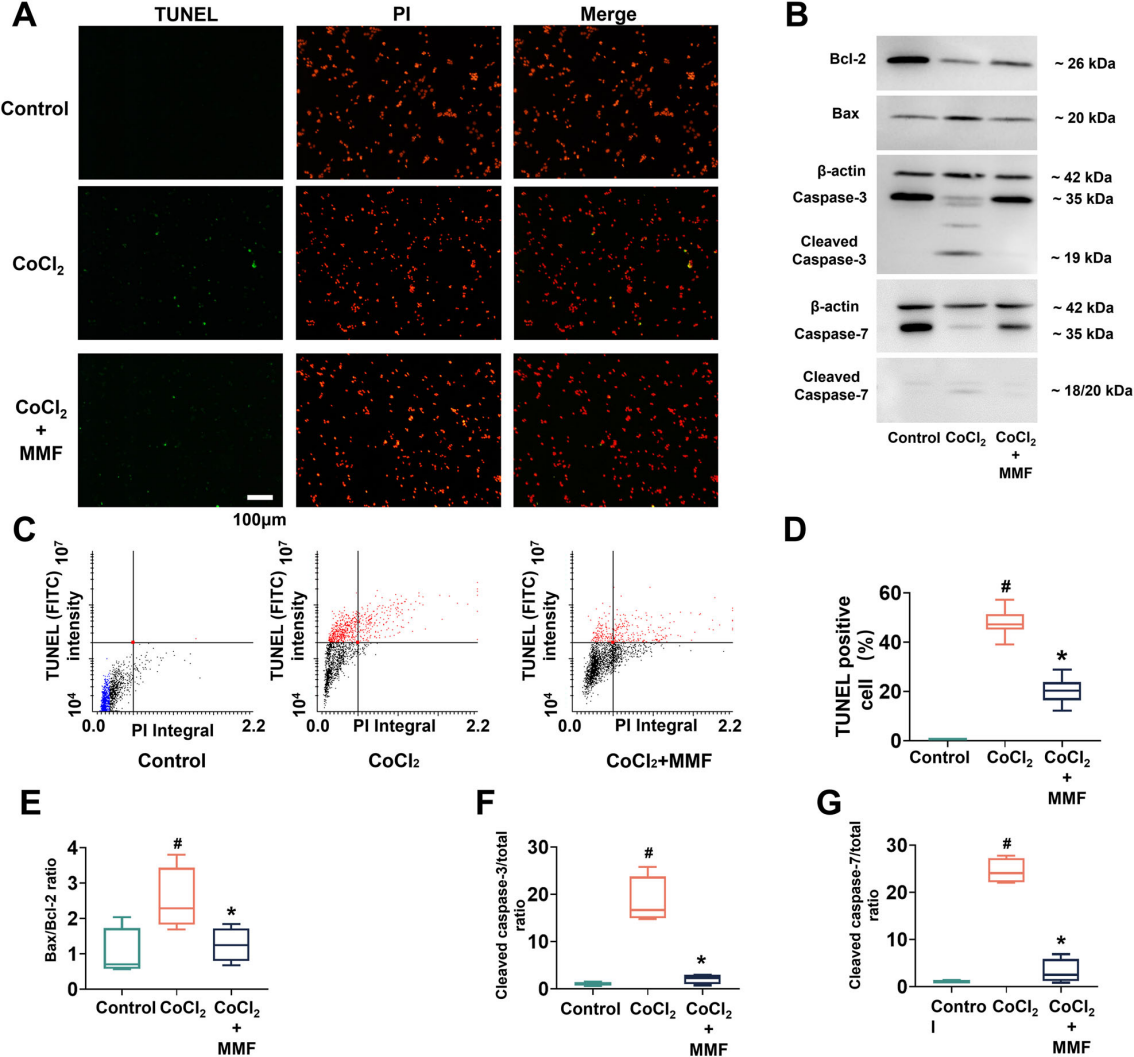
MMF prevents hypoxia-induced apoptosis in cultured HL-1 cardiomyocytes. **A** Representative fluorescence images of HL-1 cardiomyocytes under normoxic control vs hypoxic conditions (CoCl₂ treatment) ± MMF. TUNEL assay (green) marks apoptotic nuclei; propidium iodide (PI, red) counterstains all nuclei. Control cells show no apoptosis (no green nuclei). CoCl₂ (chemical hypoxia) causes many nuclei to become TUNEL-positive (green), indicating extensive apoptosis. CoCl₂ + MMF markedly reduces the number of TUNEL-positive nuclei, demonstrating an anti-apoptotic effect of MMF. Scale bar = 100 µm. **B** Western blot analysis of apoptosis-related proteins in cardiomyocyte lysates for the indicated treatments (representative blots). CoCl₂ alone decreases Bcl-2 and increases Bax relative to control, and induces cleavage of caspase-3 and caspase-7 (appearance of cleaved fragments at ~19 kDa and ~18 kDa, respectively). MMF co-treatment reverses these changes: Bcl-2 is upregulated, Bax is lower, and cleaved caspase-3/7 levels are reduced compared to CoCl₂ alone. β-Actin serves as a loading control (shown for respective blot sections)**. C** Laser Scanning Cytometry of TUNEL assay results in cardiomyocytes (PI intensity vs FITC-TUNEL). In control cells, virtually no cells fall in the TUNEL-positive region (lower-left, blue events). CoCl₂ treatment shifts a large cell population into the TUNEL-positive quadrant (red dots, indicating DNA fragmentation). CoCl₂ + MMF shows a smaller TUNEL-positive population (fewer red events), consistent with reduced apoptosis. **D** Quantification of TUNEL-positive cells (% of total) by Laser Scanning Cytometry (bar graph). CoCl₂ induces apoptosis in ~60% of cells (*p* < 0.05 vs Control), whereas MMF co-treatment lowers this to ~30% (significantly less than CoCl₂, *p < 0.05). **E**–**G** Quantitative analysis of apoptosis protein ratios from Western blots (mean ± SD). **E** Bax/Bcl-2 ratio, **F** cleaved caspase-3/caspase-3 ratio, and **G** cleaved caspase-7/caspase-7 ratio for Control, CoCl₂, and CoCl₂ + MMF groups. CoCl₂ causes a pro-apoptotic shift (higher Bax/Bcl-2 and caspase cleavage ratios vs Control, *p* < 0.05). MMF treatment significantly counteracts this shift, yielding lower Bax/Bcl-2 and caspase activation ratios compared to CoCl₂ (*p* < 0.05). Together, these data demonstrate that MMF directly protects cardiomyocytes from hypoxic injury by suppressing the apoptotic pathway. *# p* < 0.05 vs Control; *p < 0.05 vs CoCl₂. MMF monomethyl fumarate, TUNEL Terminal deoxynucleotidyl transferase dUTP Nick-End Labeling.

To directly test the effect of MMF on cardiomyocyte survival and probe mechanisms, we employed an in vitro hypoxia model. Cultured HL-1 cells were exposed to cobalt chloride (CoCl₂), a chemical hypoxia mimetic that stabilizes HIF-1α and induces an oxygen-deprivation response, in the presence or absence of MMF. Figure 5A shows fluorescence micrographs of cells under each condition (Control, CoCl₂, CoCl₂ + MMF). TUNEL staining (green) identifies apoptotic nuclei, while propidium iodide (PI, red) labels all cell nuclei. Control cells (normoxia) showed virtually no TUNEL-positive nuclei (no apoptosis). CoCl₂ exposure caused extensive apoptosis—numerous nuclei stained green, indicating DNA fragmentation. MMF treatment markedly attenuated this effect: cultures treated with CoCl₂ + MMF had far fewer TUNEL-positive cells than with CoCl₂ alone. The merged images make clear that MMF preserved the majority of cells from undergoing apoptosis under hypoxic stress. A TUNEL assay analyzed by Laser Scanning Cytometry provided quantitative confirmation (Fig. 5B). Dot plots of PI intensity vs FITC (TUNEL) signal distinguish apoptotic (TUNEL⁺, red) from non-apoptotic (black) cells. In control cells, <1% fell into the TUNEL-positive gate. CoCl₂ caused a dramatic increase in the apoptotic fraction (red cluster), whereas adding MMF shifted the distribution back towards the control profile (fewer red events). Figure 5C summarizes the TUNEL-positive cell percentages: CoCl₂ induced apoptosis in ~55–60% of the cell population (*p* < 0.05 vs control), and MMF co-treatment lowered this to ~30% (# *p* < 0.05 vs CoCl₂). Thus, MMF roughly halved the incidence of apoptosis caused by hypoxic injury, in line with the protection observed in vivo. Western blot analyses of the apoptotic pathway (Fig. 5D) further support this cytoprotection. CoCl₂-treated cells showed a decrease in Bcl-2 and an increase in Bax protein levels relative to control, mirroring the pro-apoptotic shift seen in vivo. In addition, CoCl₂ markedly activated caspases, as evidenced by the appearance of cleaved (active) caspase-3 and caspase-7 fragments on the blots. MMF treatment largely reversed these molecular changes: Bcl-2 levels were higher with MMF, Bax and cleaved caspase-3/7 levels were lower, compared to CoCl₂ alone. Quantitative data (Fig. 5E–H) demonstrate that MMF significantly normalized the Bax/Bcl-2 ratio and the cleaved caspase/total caspase ratios. For example, CoCl₂ elevated the Bax/Bcl-2 ratio ~3-fold above control (*p* < 0.05), whereas CoCl₂ + MMF brought the ratio down closer to normal (# *p* < 0.05 vs CoCl₂) (Fig. 5F). Similarly, the cleaved caspase-3 to procaspase-3 ratio was greatly increased by CoCl₂ (reflecting caspase-3 activation), and this was significantly reduced with MMF co-treatment (Fig. 5G). Cleaved caspase-7 showed the same pattern (Fig. 5H). These in vitro results show that MMF can directly protect cardiomyocytes from hypoxia-induced apoptosis, paralleling the in vivo findings, and they set the stage for dissecting the signaling mechanisms involved.

### HCAR2 is required for the anti-apoptotic effect of MMF

**Fig. 6:**
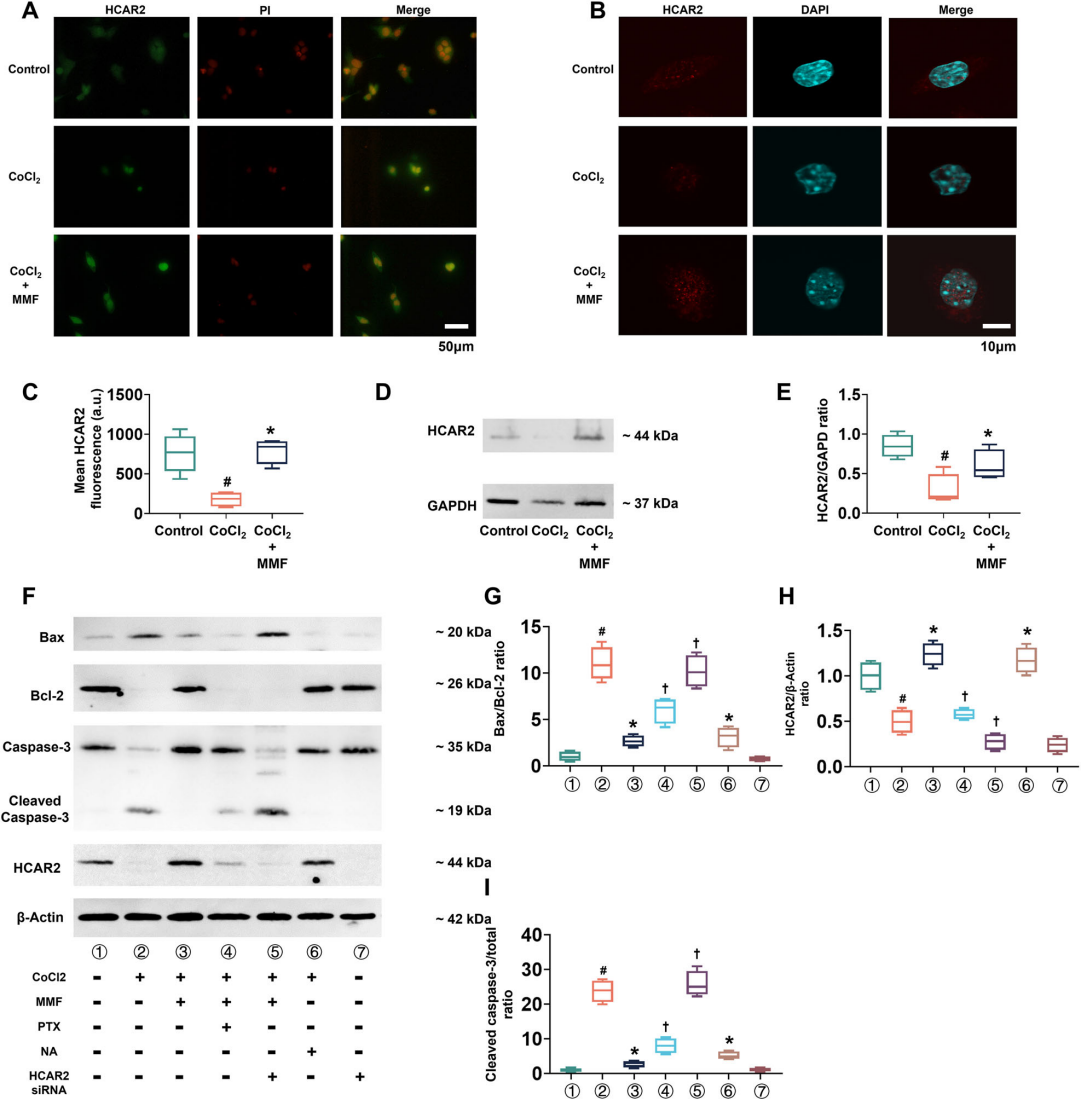
MMF’s protective effect requires HCAR2 receptor signaling in cardiomyocytes. **A** Immunofluorescence staining of HCAR2 in cultured cardiomyocytes under various treatments: Control (normoxia), CoCl₂ (hypoxia), and CoCl₂ + MMF. In these images, HCAR2 is shown in green and nuclei are counterstained with propidium iodide (PI, red). Control cells exhibit strong HCAR2 expression (green) in the cytoplasm/membrane. CoCl₂-treated cells show a drastic loss of HCAR2 signal (very faint green), indicating downregulation of HCAR2 during hypoxia. CoCl₂ + MMF cells retain higher HCAR2 intensity (more green signal visible) compared to CoCl₂ alone, suggesting MMF preserves HCAR2 expression. Scale bar = 50 µm. **B** High-magnification confocal images of HCAR2 localization (red) in individual cardiomyocytes, with DAPI nuclear stain (blue). Control cells have abundant punctate and membranous HCAR2 staining. Under CoCl₂, HCAR2 signal is nearly absent. With MMF, HCAR2 signal is partially restored in the cells. Scale bar = 10 µm. **C** Mean fluorescence intensity of HCAR2 per cell (arbitrary units) under each condition. CoCl₂ causes an ~80% reduction in HCAR2 intensity vs Control (*p* < 0.05), whereas MMF co-treatment significantly increases HCAR2 signal relative to CoCl₂ (*p* < 0.05). **D** Western blot for HCAR2 protein (44 kDa) in Control, CoCl₂, and CoCl₂ + MMF groups (GAPDH as loading control). HCAR2 protein is abundant in control cells, sharply reduced with CoCl₂, and partially recovered in the MMF-treated cells. **E** Densitometry of HCAR2/GAPDH ratio from blots (mean ± SD). CoCl₂ decreases HCAR2 to ~20% of control levels (*p* < 0.05), while MMF-treated cells show ~50% of control HCAR2, significantly higher than CoCl₂ alone (*p* < 0.05). **F** Western blots examining the necessity of HCAR2 in MMF’s anti-apoptotic effect. Cells were subjected to CoCl₂ with or without MMF, in the presence of HCAR2 pathway inhibitors: pertussis toxin (PTX, a Gi protein inhibitor) or HCAR2-targeted siRNA knockdown. Blots for pro- and anti-apoptotic proteins are shown: BAX, BCL-2, full-length caspase-3 (35 kDa) and cleaved caspase-3 (19 kDa), as well as HCAR2 (to verify knockdown) and β-actin (42 kDa) as loading control. Lane treatments are:① Control; ②CoCl₂; ③ CoCl₂ + MMF; ④ CoCl₂ + PTX; ⑤ CoCl₂ + MMF + PTX; ⑥ CoCl₂ + HCAR2 siRNA; ⑦ CoCl₂ + MMF + HCAR2 siRNA. CoCl₂ (lane 2) increases BAX and cleaved caspase-3 while reducing BCL-2, relative to control. MMF (lane 3) reverses these changes (lower BAX and cleaved caspase-3, higher BCL-2). Importantly, when HCAR2 is blocked—by PTX (lane 5) or by HCAR2 knockdown (lane 7)—the MMF effect is lost: BAX and cleaved caspase-3 remain high and BCL-2 remains low, similar to CoCl₂ alone (lane 2). Lanes 6–7 confirm successful HCAR2 knockdown (HCAR2 band is absent in siRNA-treated cells). **G**–**I** Quantification of apoptotic indicators across the seven treatment conditions (corresponding to lanes 1–7 in **F**). **G** Bax/Bcl-2 ratio; **H** HCAR2/β-actin protein level; **I** Cleaved caspase-3/caspase-3 ratio. CoCl₂ dramatically elevates the Bax/Bcl-2 and caspase-3 cleavage ratios vs control (*p* < 0.05). MMF significantly lowers both ratios compared to CoCl₂ (*p* < 0.05). However, with PTX or HCAR2 knockdown, the ratios remain high (no significant reduction vs CoCl₂), indicating that blocking HCAR2 signaling abolishes MMF’s ability to suppress apoptosis. (H) shows HCAR2 protein is effectively silenced by siRNA (near-zero in conditions 6–7). All data are mean ± SD; # *p* < 0.05 vs Control; * *p* < 0.05 vs CoCl₂ alone; for (**G**–**H**), †*p* < 0.05vs CoCl₂ + MMF. These results establish HCAR2 as a requisite mediator of MMF’s anti-a*p*optotic action in cardiomyocytes. MMF monomethyl fumarate, TUNEL Terminal deoxynucleotidyl transferase dUTP Nick-End Labeling, DAPI 4’,6-diamidino-2-phenylindole, HCAR2 Hydroxycarboxylic Acid Receptor 2, GAPDH glyceraldehyde-3-phosphate dehydrogenase, PTX pertussis toxin (Gi inhibitor), NA nicotinic acid, an HCAR2 agonist.

We hypothesized that MMF’s protective action is mediated through the HCAR2 receptor. To test this, we examined HCAR2 expression in our cell model and used pharmacological and genetic approaches to block HCAR2 signaling. First, we confirmed that CoCl₂ indeed downregulates HCAR2 in cardiomyocytes, as observed in vivo. Figure 6A shows immunofluorescence staining of cultured cardiomyocytes for HCAR2 (green) with nuclear PI counterstain (red). Control cells displayed clear HCAR2 staining (green) in the cytoplasm/membrane, whereas in hypoxic (CoCl₂) cells HCAR2 fluorescence was barely detectable. In the CoCl₂ + MMF group, HCAR2 signal intensity was restored to a level noticeably above that of CoCl₂-only cells, indicating that MMF prevented the loss of HCAR2 protein under hypoxic stress. Higher-magnification confocal images (Fig. 6B, HCAR2 in red, nuclei in blue) reinforced this finding: intense HCAR2 staining is evident in control cells, nearly absent in CoCl₂-treated cells, and partially preserved in the presence of MMF. Quantification of mean HCAR2 fluorescence per cell (Fig. 6C) showed a >80% drop in HCAR2 intensity with CoCl₂ relative to control (*p* < 0.05), whereas cells co-treated with MMF had roughly double the HCAR2 signal of CoCl₂-only cells (# *p* < 0.05 vs CoCl₂). Consistent with the imaging, Western blotting confirmed that CoCl₂ markedly reduced HCAR2 protein levels, and MMF rescued this expression. Figure 6D presents HCAR2 immunoblots for each condition: the HCAR2 band (∼44 kDa) is strong in control, virtually gone with CoCl₂, and partially restored with MMF. Densitometry (Fig. 6E) revealed HCAR2/GAPDH protein ratio dropping to ~20% of control in CoCl₂-treated cells (*p* < 0.05), while in CoCl₂ + MMF cells it was ~50% of control (# *p* < 0.05 vs CoCl₂). Having established that MMF prevents hypoxia-induced HCAR2 loss, we next asked if HCAR2 activity is *necessary* for MMF’s anti-apoptotic effect. We inhibited HCAR2 signaling by two independent means: (1) using pertussis toxin (PTX) to block Gi/o-protein coupled receptors (HCAR2 signals via Gi), and (2) using siRNA to knock down HCAR2 gene expression. Figure 6F shows Western blots of apoptosis regulators under these various conditions. The seven lanes correspond to: (1) control; (2) CoCl₂; (3) CoCl₂ + MMF; (4) CoCl₂ + PTX; (5) CoCl₂ + MMF + PTX; (6) CoCl₂ + HCAR2-siRNA; (7) CoCl₂ + MMF + HCAR2-siRNA. As expected, lane 2 (CoCl₂) shows an apoptosis-prone profile (high Bax, low Bcl-2, high cleaved caspase-3) relative to control (lane 1), and lane 3 (CoCl₂ + MMF) shows a shift toward survival (lower Bax, higher Bcl-2, less cleaved caspase-3). Critically, when HCAR2 was blocked, MMF could no longer enforce this shift. In lane 5 (MMF + PTX) and lane 7 (MMF + HCAR2 knockdown), the protein levels resemble those of CoCl₂ alone: Bax remains elevated, Bcl-2 remains low, and cleaved caspase-3 is abundant—indicating loss of MMF’s protective effect. The necessity of HCAR2 is quantified in Fig. 6G–I. CoCl₂ increased the Bax/Bcl-2 ratio ~5-fold vs control (*p* < 0.05), and MMF (lane 3) significantly lowered this ratio (# *p* < 0.05 vs CoCl₂). However, in HCAR2-blocked conditions (lanes 5 and 7), the Bax/Bcl-2 ratio remained high (no significant reduction vs CoCl₂ alone, Fig. 6G). Similarly, MMF failed to reduce the cleaved caspase-3/total caspase-3 ratio when HCAR2 was inhibited: as shown in Fig. 6I, the low caspase-3 activation seen with MMF (lane 3) was abolished by PTX or HCAR2 knockdown (lanes 5, 7 had caspase cleavage levels comparable to CoCl₂). We also confirmed effective HCAR2 knockdown: in Fig. 6H, the HCAR2/β-actin ratio was near zero in siRNA-treated cells (lanes 6, 7) versus robust HCAR2 expression in non-silenced conditions. These results demonstrate that HCAR2 is essential for MMF’s anti-apoptotic action in cardiomyocytes. In the absence of HCAR2 signaling, MMF no longer confers protection against hypoxic injury.

**Fig. 7:**
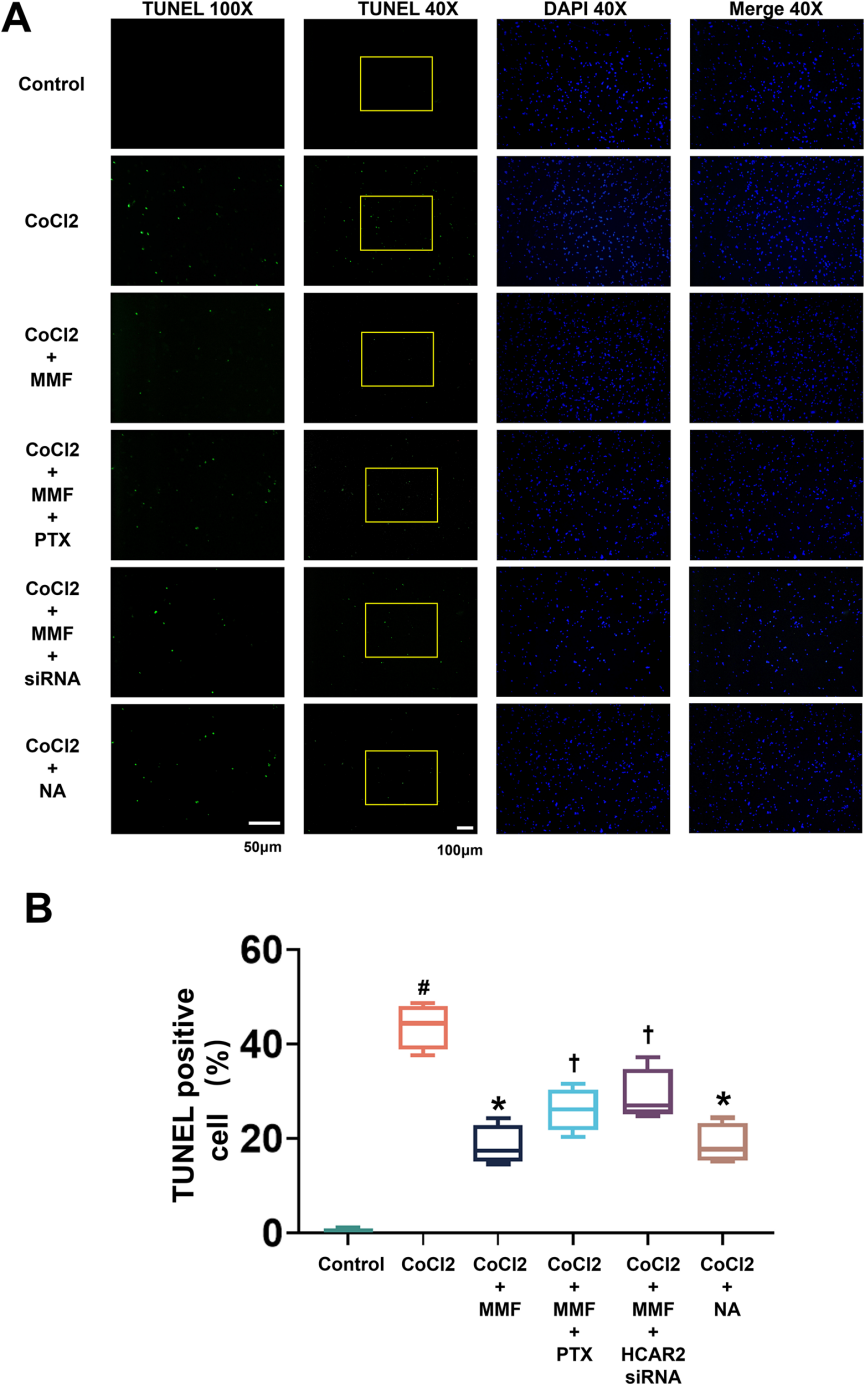
TUNEL assay confirms HCAR2 dependence of MMF’s anti-apoptotic effect (and is mimicked by HCAR2 agonist). **A** Representative images of TUNEL-stained cardiomyocytes (green = TUNEL, blue = DAPI) under the following conditions: Control, CoCl₂, CoCl₂ + MMF, CoCl₂ + MMF + PTX, CoCl₂ + MMF + HCAR2 siRNA, and CoCl₂ + nicotinic acid (NA, 1 mM). For each condition, a low-magnification overview (40×) and a higher magnification inset (100×, corresponding to the yellow boxed region) are shown to visualize apoptotic nuclei. Control cells show no TUNEL positivity. CoCl₂ causes abundant TUNEL-positive nuclei (green) throughout the field, indicating extensive apoptosis. CoCl₂ + MMF shows markedly fewer apoptotic cells (sparse green nuclei). However, when MMF is combined with PTX or HCAR2 knockdown, many TUNEL-positive nuclei reappear (comparable to CoCl₂ alone), demonstrating loss of MMF’s protection. Treatment with nicotinic acid (CoCl₂ + NA), an HCAR2 agonist, also results in a low number of TUNEL-positive cells, similar to MMF’s effect. Scale bars: 40× images = 100 µm; 100× images = 50 µm. **B** Quantitation of TUNEL-positive cells (% of total) for each condition (box-and-whisker plot). CoCl₂ induces ~50–60% of cells to undergo apoptosis (#*p* < 0.05 vs Control). MMF co-treatment reduces this to ~20% (*p < 0.05 vs CoCl₂). The addition of PTX or HCAR2 siRNA significantly blunts MMF’s effect, raising apoptosis to ~30–35% († *p* < 0.05 vs CoCl₂ + MMF; still lower than CoCl₂ alone, *p* < 0.05 vs CoCl₂ in the case of PTX). Nicotinic acid treatment (CoCl₂ + NA) also yields a low apoptosis rate (~25%, *p* < 0.05 vs CoCl₂). These results mirror the Western blot data and demonstrate that MMF’s anti-apoptotic benefit is nullified by HCAR2 inhibition, while direct activation of HCAR2 by NA can independently protect cells. *# p* < 0.05 vs Control; *p < 0.05 vs CoCl₂; † *p* < 0.05 vs CoCl*₂* + MMF (as indicated by comparison bars in graph). MMF monomethyl fumarate, TUNEL Terminal deoxynucleotidyl transferase dUTP Nick-End Labeling, DAPI 4’,6-diamidino-2-phenylindole; HCAR2, Hydroxycarboxylic Acid Receptor 2, PTX pertussis toxin (Gi inhibitor), NA nicotinic acid, an HCAR2 agonist.

To further support the role of HCAR2, we examined apoptosis using TUNEL assays under the same conditions. Figure 7A shows representative images of TUNEL-stained cardiomyocytes for each treatment group (green = TUNEL, blue = DAPI). Consistent with earlier data, CoCl₂ induced massive apoptosis (~ green nuclei throughout), which was clearly reduced by MMF. However, when MMF was combined with PTX or HCAR2 siRNA, the incidence of TUNEL-positive cells remained high (comparable to CoCl₂ alone). In contrast, adding nicotinic acid (NA, 1 mM)—a direct HCAR2 agonist—to CoCl₂-treated cells mimicked the effect of MMF, resulting in markedly fewer TUNEL-positive nuclei. Quantitative analysis (Fig. 7B) showed ~50% TUNEL-positive cells with CoCl₂, decreasing to ~20% with MMF (*p* < 0.05 vs CoCl₂). PTX or HCAR2 knockdown significantly blunted the reduction (apoptosis levels climbed back to ~30–35% with either intervention, *p* < 0.05 vs CoCl₂ + MMF). Meanwhile, CoCl₂ + NA yielded ~25% TUNEL-positive cells, a significant improvement vs CoCl₂ (*p* < 0.05). These findings strongly indicate that activating HCAR2 is both necessary and sufficient to achieve the anti-apoptotic effect—MMF requires HCAR2 to protect cells, and direct HCAR2 stimulation by an agonist can replicate the protection. This aligns with the known biology of HCAR2 as a receptor capable of modulating cell survival and inflammation [9]. Indeed, niacin/HCAR2 activation has been reported to induce anti-inflammatory or protective responses in other contexts [9], which our data now extend to cardiomyocyte survival after hypoxic stress.

### Activation of the PI3K/Akt pathway is necessary for MMF-mediated cytoprotection

**Fig. 8:**
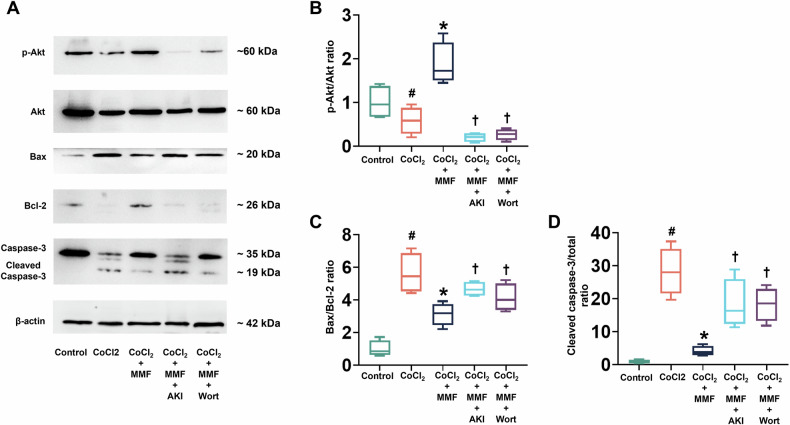
MMF’s anti-apoptotic effect is mediated via the PI3K/Akt signaling pathway. **A** Western blot analysis of the PI3K/Akt dependence of MMF’s protection in cardiomyocytes. Five treatment conditions are shown: Control (no CoCl₂), CoCl₂ alone, CoCl₂ + MMF, CoCl₂ + MMF + Akt inhibitor (AKi, 10 µM), and CoCl₂ + MMF + Wortmannin (Wort, PI3K inhibitor, 100 nM). Blots for phosphorylated Akt (Ser473) and total Akt confirm pathway activation or inhibition; blots for Bax, Bcl-2, and caspase-3 (full-length ~35 kDa, and cleaved ~19 kDa) show apoptosis markers; β-actin is a loading control. CoCl₂ alone markedly reduces p-Akt levels relative to control, indicating suppressed Akt activity under hypoxia. MMF co-treatment greatly increases p-Akt (lane 3), consistent with activation of Akt. MMF also upregulates Bcl-2 and reduces Bax and cleaved caspase-3 compared to CoCl₂ alone. In the presence of Akt/PI3K inhibitors (lanes 4–5), MMF can no longer induce these changes: p-Akt is nearly absent (confirming successful pathway blockade) and the protein expression pattern (high Bax, low Bcl-2, strong caspase-3 cleavage) resembles that of CoCl₂ alone. **B**–**D** Quantitative comparisons of key apoptosis-related ratios across the conditions (mean ± SD). **B** p-Akt/Akt ratio. CoCl₂ significantly decreases Akt phosphorylation vs Control (#*p* < 0.05, *n* = 4). MMF raises p-Akt/Akt ~2–3-fold vs CoCl₂ (*p < 0.05, *n* = 4). The Akt inhibitor and Wortmannin prevent this increase, keeping p-Akt/Akt at or below the CoCl₂ baseline († *p* < 0.05 vs CoCl₂ + MMF). **C** Bax/Bcl-2 ratio. CoCl₂ causes a large increase in Bax/Bcl-2 vs Control (*p* < 0.05, *n* = 4), whereas MMF brings the ratio down significantly (*n* = 4 * *p* < 0.05 vs CoCl₂, indicating restoration of a more anti-apoptotic balance). With Akt or PI3K inhibited, the Bax/Bcl-2 ratio rises again (†*p* < 0.05 vs MMF; no longer different from CoCl₂ alone), showing loss of MMF’s effect. **D** Cleaved caspase-3/total caspase-3 ratio. This index of caspase-3 activation is elevated ~10-fold by CoCl₂ vs Control (#*p* < 0.05, *n* = 4). MMF treatment cuts the caspase-3 activation roughly in half (*p < 0.05 vs CoCl₂, *n* = 4). However, when Akt signaling is blocked, cleaved caspase-3 levels revert toward the high CoCl₂ value († *p* < 0.05 vs CoCl₂ + MMF, *n* = 4), indicating that MMF can no longer suppress caspase activation. Collectively, these results demonstrate that active PI3K/Akt signaling is required for MMF’s anti-apoptotic, cardioprotective action. #*p* < 0.05 vs Control; *p < 0.05 vs CoCl₂; † *p* < 0.05 vs CoCl₂ + MMF. MMF monomethyl fumarate, AKI Akt inhibitor IV, a specific Akt inhibitor drug, Wort Wortmannin, a specific PI3K inhibitor drug.

Given the observed changes in Akt phosphorylation with MMF, we next examined whether the PI3K/Akt signaling pathway is a critical mediator of MMF’s cytoprotective effect. Akt is a well-known pro-survival kinase that can inhibit apoptosis by phosphorylating and inactivating key components of the apoptotic machinery (e.g., caspase-9, Bax, Bad) [10]. We therefore tested if blocking Akt signaling would negate MMF’s benefits. Figure 8A shows Western blots of cardiomyocytes under five conditions: control, CoCl₂, CoCl₂ + MMF, CoCl₂ + MMF + Akt inhibitor (AKi), and CoCl₂ + MMF + Wortmannin (a PI3K inhibitor). As previously seen, CoCl₂ alone (lane 2) drastically reduced p-Akt levels compared to control (lane 1), while MMF treatment (lane 3) strongly increased p-Akt (Ser473) despite continued CoCl₂ exposure. Correspondingly, MMF (lane 3) maintained higher Bcl-2 and lower Bax and cleaved caspase-3 levels relative to CoCl₂ alone. Strikingly, both the Akt inhibitor and Wortmannin abolished these MMF-induced effects. In lanes 4 and 5, p-Akt was suppressed (nearly undetectable), and the apoptosis markers reverted toward a pro-death profile: Bax was up-regulated, Bcl-2 down, and cleaved caspase-3 abundant—essentially mirroring the CoCl₂-only pattern. Thus, when the PI3K/Akt pathway was inhibited, MMF could no longer protect the cells. The quantitative analysis in Fig. 8B–D reinforces this conclusion. Akt activation (p-Akt/Akt ratio, Fig. 8B) was significantly modulated by our treatments: CoCl₂ reduced p-Akt/Akt to ~0.8 (from ~1.3 in control, *p* < 0.05), MMF boosted it to ~2.2 (# *p* < 0.05 vs CoCl₂), but adding AKi or Wortmannin brought p-Akt/Akt down to ~0.5–0.6, which was not only far below the MMF value (†*p* < 0.05 vs CoCl₂ + MMF) but even below the CoCl₂-only level. Meanwhile, the Bax/Bcl-2 ratio (Fig. 8C) rose sharply with CoCl₂ (*p* < 0.05 vs control), dropped with MMF (# *p* < 0.05 vs CoCl₂), and climbed back up when Akt was blocked († *p* < 0.05 vs CoCl₂ + MMF; no significant difference between CoCl₂ + AKi or Wort and CoCl₂ alone). A similar trend was evident for the cleaved caspase-3/caspase-3 ratio (Fig. 8D): MMF significantly suppressed caspase-3 activation vs CoCl₂ (# *p* < 0.05), but in the presence of PI3K/Akt inhibitors the cleaved caspase-3 levels returned toward the high values seen with CoCl₂ (*p* < 0.05 vs control, †*p* < 0.05 vs MMF group). Together, these results demonstrate that the PI3K/Akt pathway is indispensible for MMF’s protective effect. MMF appears to rescue cells by turning on Akt signaling, and when this pathway is chemically disabled, cell death is no longer prevented. This is consistent with extensive evidence that Akt activation promotes cardiomyocyte survival and mitigates post-infarct cardiac injury [10]. Indeed, activating the PI3K/Akt cascade has been shown to alleviate adverse remodeling after MI and reduce cardiomyocyte apoptosis in multiple models [10]. Our findings place Akt as a key downstream effector of HCAR2 in the context of MMF therapy.

## Discussion

In this study, we identify MMF as a potent cardioprotective agent in the setting of acute myocardial infarction. MMF administration (prior to ischemia in our model) led to striking preservation of cardiac function and a reduction in cardiomyocyte apoptosis after MI. Mechanistically, our data reveal that MMF’s protective effects are mediated through the HCAR2 receptor and activation of the PI3K/Akt signaling pathway. To our knowledge, this is the first demonstration that HCAR2—a receptor previously studied in immunological and metabolic contexts—plays a critical role in protecting the heart from ischemic injury. These findings not only shed light on MMF’s mechanism of action in the cardiovascular system but also suggest a novel therapeutic approach for ischemic heart disease by targeting HCAR2.

Comparison with previous studies: Our results extend the growing body of literature on the cardiovascular benefits of fumarate compounds. Prior studies with DMF (the prodrug of MMF) have shown protective effects in various models of cardiac injury, largely attributed to its antioxidant and anti-inflammatory properties [1]. For example, Meili-Butz et al. reported that DMF reduced infarct size in a rat ischemia-reperfusion model, an effect linked to inhibition of NF-κB and reduced inflammatory cytokine expression [1, 2]. Another study demonstrated that DMF preserves post-MI cardiac structure and function when given in the subacute phase: Mouton et al. found that 10 mg/kg DMF administered for 5 days after MI improved ventricular wall thickness, increased infarct-zone angiogenesis, and suppressed inflammatory gene expression in mice [1]. These benefits were associated with elevated Nrf2 levels and a metabolic shift in cardiac macrophages toward oxidative phosphorylation [1]. Our current study aligns with these reports on the end-effect (improved cardiac outcomes) but provides new mechanistic insight by pinpointing a specific receptor and signaling pathway involved. Notably, much of the prior work has emphasized the *Nrf2 pathway* as central to DMF/MMF action [1, 3]. In isolated cardiomyocytes and H9c2 cells, DMF was shown to activate Nrf2, leading to upregulation of antioxidant enzymes (HO-1, NQO1) and reduction of ROS and apoptosis [1, 3]. Our findings do not contradict the Nrf2-mediated antioxidant mechanism; rather, they reveal an additional, complementary mechanism: HCAR2-dependent PI3K/Akt activation. It is intriguing that MMF can operate via HCAR2 to promote cell survival, whereas Nrf2 activation by MMF might occur through direct glutathione depletion and electrophilic stress (independently of HCAR2) [3]. Indeed, a recent study in cardiac I/R by Kuang et al. demonstrated that DMF’s protection was abrogated in Nrf2-deficient conditions [3], confirming Nrf2’s importance. We suspect that in vivo, MMF engages multiple pathways: an intrinsic cellular stress response (Nrf2) and extrinsic receptor signaling (HCAR2).

The identification of HCAR2 as a mediator of MMF’s cardiac effects is novel. HCAR2 is best known as the receptor for niacin; its activation on skin Langerhans cells causes flushing, and on adipocytes it inhibits lipolysis. However, HCAR2 also modulates immune cell function and inflammation [4]. In the context of neuroinflammation, Parodi et al. found that MMF’s neuroprotective effect required HCAR2 and entailed a downstream cascade (AMPK–Sirt1–NF-κB) that reprogrammed microglia to an anti-inflammatory state [4]. By analogy, in the infarcted heart, HCAR2 activation could be exerting beneficial immunomodulation—for instance, by dampening neutrophil and macrophage activity known to exacerbate reperfusion injury [2]. Our observation that HCAR2 expression was preserved or upregulated by MMF in ischemic myocardium hints that MMF may counteract an MI-induced loss of HCAR2. It is possible that ischemia and inflammatory mediators downregulate HCAR2 on cardiac cells or infiltrating leukocytes; MMF might prevent this or even induce HCAR2 expression on certain cells (potentially through Nrf2 or other transcriptional changes). The functional consequence would be enhanced autocrine or paracrine HCAR2 signaling. We noted HCAR2 staining on cells at the infarct border zone, which could include macrophages or cardiomyocytes. If those cells respond to MMF via HCAR2, they may produce cytoprotective signals (e.g., anti-inflammatory cytokines like IL-10, as seen with fumarates in stroke models [2]). Although our study did not directly measure cytokine levels in vivo, MMF-treated hearts likely had a muted inflammatory response, as evidenced by less apoptosis and injury. This is consistent with DMF’s known ability to reduce IL-1β, IL-6, and TNF-α in injured tissues [1].

In cardiomyocytes specifically, how might HCAR2 activation lead to survival? Our in vitro experiments provide a clue: HCAR2 (via Gαi proteins) appears to trigger the PI3K/Akt pathway. Gαi signaling can release Gβγ subunits that activate PI3K, resulting in Akt phosphorylation. We showed that MMF fails to phosphorylate Akt when Gαi is inhibited by pertussis toxin or when HCAR2 is knocked down (thus Gαi is not engaged). This strongly suggests a linear sequence: MMF → HCAR2 → Gαi/βγ → PI3K → Akt → pro-survival effects. Akt in turn phosphorylates numerous targets to suppress apoptosis (for example, Bad, a Bcl-2 family protein, is inactivated by Akt). Indeed, we observed higher Bcl-2/Bax ratio and less caspase-3 activation in the presence of MMF, which is a downstream reflection of Akt’s pro-survival signaling. The PI3K/Akt pathway is a well-established mediator of ischemic preconditioning and many pharmacological cardioprotective agents (e.g., insulin, growth factors) [3]. Our work is the first to connect a GPCR agonist (MMF) acting on HCAR2 to this cardioprotective kinase cascade. Interestingly, niacin has been reported in some studies to exhibit anti-inflammatory effects beyond lipid lowering [9], and it raises adiponectin levels which could be cardioprotective [2], but niacin has not been widely recognized to activate Akt in the heart. It is possible that MMF is a more efficacious or context-specific HCAR2 agonist for cardiomyocytes than niacin. We noted that nicotinic acid at high concentration only partially reproduced MMF’s effect in HL-1 cells, hinting that MMF might have biased agonism or additional intracellular targets. MMF, being a small electrophilic molecule, can also modify Keap1 (to activate Nrf2) [3] and modulate glutathione—pathways niacin would not affect. Thus, MMF likely provides a dual stimulus: one via HCAR2→Akt and another via Nrf2, converging to enhance cell survival.

### Translational implications

Our findings highlight MMF (and by extension, its prodrug DMF) as a promising therapeutic candidate for acute MI. An important advantage is that DMF is already an FDA-approved drug (for multiple sclerosis), with a known safety profile in humans [4]. This accelerates the potential for repurposing DMF/MMF in cardiovascular indications. Notably, the doses of DMF used in animal studies for cardioprotection (around 10 mg/kg in rodents) translate to the lower range of human MS dosing on a mg/kg basis [1], suggesting feasibility. MMF itself could be used intravenously for rapid delivery in acute settings, avoiding the delay of prodrug conversion. One could envision administering MMF at the time of reperfusion (e.g., during PCI for ST-elevation MI) to reduce reperfusion injury. In this regard, it is encouraging that therapies targeting inflammation have shown benefits in post-MI patients: the CANTOS trial demonstrated that an IL-1β neutralizing antibody reduced recurrent cardiovascular events [11], and the COLCOT trial showed low-dose colchicine (an anti-inflammatory drug) lowered the risk of ischemic complications after MI [12]. MMF/DMF, with their combined anti-inflammatory and cytoprotective actions, could potentially offer both immediate myocardial salvage and longer-term remodeling benefits. Moreover, unlike highly targeted agents such as canakinumab, MMF acts on broad endogenous protective pathways (Nrf2, HCAR2/Akt) which might confer multi-faceted benefits (antioxidant, anti-apoptotic, pro-angiogenic, etc.) [1]. That said, any clinical application must consider DMF’s side effect profile—particularly flushing and gastrointestinal symptoms. The flushing is mediated by HCAR2 activation on skin cells causing prostaglandin release, a mechanism shared by niacin [4]. While flush can be mitigated (e.g., with aspirin or slow uptitration), it does indicate that systemic HCAR2 engagement has pharmacodynamic effects. In acute MI patients who are often critically ill, flushing or transient hypotension (from vasodilatation) could be undesirable. Therefore, an alternative strategy might be to design HCAR2-biased agonists that preferentially trigger intracellular cardioprotective signaling without strong prostaglandin release. The structural biology of HCAR2 is being elucidated [13, 14], which could aid rational design of such ligands.

### Limitations

Several limitations of our study should be acknowledged. First, our MMF treatment was given prophylactically (before ischemia in mice and before simulated hypoxia in cells). This approach, while useful for proof-of-concept, does not reflect the clinical scenario where a therapy would be started at the time of or after an acute MI. Prophylactic administration in patients is generally impractical unless in high-risk situations (e.g., before cardiac surgery or angioplasty in unstable angina). It will be important in future studies to determine whether MMF confers cardioprotection when given at reperfusion or post-MI. Some prior work suggests DMF can be effective even when started after MI [1], which is encouraging. Second, our cellular model utilized HL-1 cardiomyocytes and CoCl₂ to induce hypoxia. HL-1 cells, while exhibiting many cardiomyocyte properties, are an immortalized atrial cell line and may not perfectly represent adult ventricular myocytes’ response to ischemia. In addition, CoCl₂ creates a hypoxia-like response mainly by stabilizing HIF-1α; it may not recapitulate all aspects of ischemia-reperfusion injury (such as abrupt pH shifts, Ca2+ overload upon reoxygenation, etc.). We chose CoCl₂ for simplicity and reproducibility, but complementary models (e.g., true oxygen-glucose deprivation followed by reoxygenation in cardiomyocytes, or ex vivo perfused heart I/R models) would strengthen the evidence. Third, while we showed HCAR2 is required for MMF’s protective effect in vitro, we did not directly prove this in vivo (e.g., using HCAR2-knockout mice or an HCAR2 antagonist in the MI model). Our in vivo data are associative—MMF preserved HCAR2 levels and activated Akt, and overall outcomes were better. It would be informative to treat HCAR2(-/-) mice with MMF during MI to confirm that the in vivo benefits indeed depend on HCAR2. This experiment is a logical next step. Fourth, we focused on apoptosis and acute injury; thus, we only evaluated hearts at 5 days post-MI. Long-term effects of MMF on post-MI remodeling (fibrosis, hypertrophy, and cardiac function over weeks) were not assessed here. Given DMF’s effects on chronic remodeling reported by others [1], it is likely that MMF could favorably influence scar formation and ventricular dilation if therapy is continued. Future studies should address the chronic phase, possibly including endpoints like infarct size by histology, fibrotic scar measurements, and ejection fraction at later time points. Finally, while our data suggest a model wherein cardiomyocyte HCAR2 activation drives Akt signaling, the involvement of other cell types is not excluded. HCAR2 on infiltrating immune cells might modulate the inflammatory milieu in ways that indirectly benefit cardiomyocytes (for example, enhancing the clearance of debris or reducing neutrophil protease release). Distinguishing direct cardiomyocyte-protective effects from systemic or paracrine effects would require cell-specific knockouts (cardiomyocyte-specific HCAR2 deletion vs. myeloid cell HCAR2 deletion). Such studies could delineate whether the primary locus of protection is within the cardiomyocyte or external to it.

Future directions: Building on our findings, several avenues merit exploration. (1) *Therapeutic window:* Determine the efficacy of MMF when administered at clinically relevant time points (on reperfusion or even after MI onset). This could involve post-conditioning experiments in animals or using DMF (oral) as a post-MI treatment in translational models. (2) *HCAR2 targeting:* Investigate other HCAR2 agonists or modulators in MI. For instance, evaluating niacin or newer HCAR2 agonists (some are under development for dyslipidemia without flushing) could reveal if they afford similar cardioprotection. Conversely, using an HCAR2 antagonist in our model would help confirm HCAR2’s role. (3) *Combination with reperfusion therapy:* Since reperfusion is standard of care, testing MMF alongside reperfusion injury models (e.g., ischemia followed by reperfusion in vivo, rather than permanent occlusion) is important. Fumarates might especially mitigate reperfusion injury (when oxidative burst and inflammation are highest) [2]. (4) *Molecular mechanisms:* Further dissection of the downstream signaling from HCAR2 in cardiomyocytes is warranted. We showed Akt is a key player; it would be interesting to see if AMPK or Sirt1 (implicated in microglial HCAR2 signaling [4]) also contribute in cardiac cells. Additionally, transcriptomic or proteomic analysis of MMF-treated hearts could uncover pathways influenced by HCAR2 activation (e.g., changes in metabolic genes, autophagy markers, etc.). (5) *Inflammation and immune response:* Future studies should measure inflammatory cell infiltration and cytokine profiles in MI ± MMF hearts. We predict MMF will reduce neutrophil infiltration and pro-inflammatory cytokines (akin to DMF’s effects in other models [1]), but this needs confirmation. Understanding how MMF influences the innate immune response after MI could strengthen the rationale for its use, especially given the current interest in anti-inflammatory strategies for cardiovascular disease [11]. (6) *Chronic remodeling:* Long-term experiments to see if MMF (or intermittent DMF dosing) post-MI can improve cardiac repair and function over weeks would be valuable. Endpoints like scar size, ejection fraction at 4 or 8 weeks, and heart failure biomarkers would determine if the acute benefits translate into sustained improvement.

**Fig. 9:**
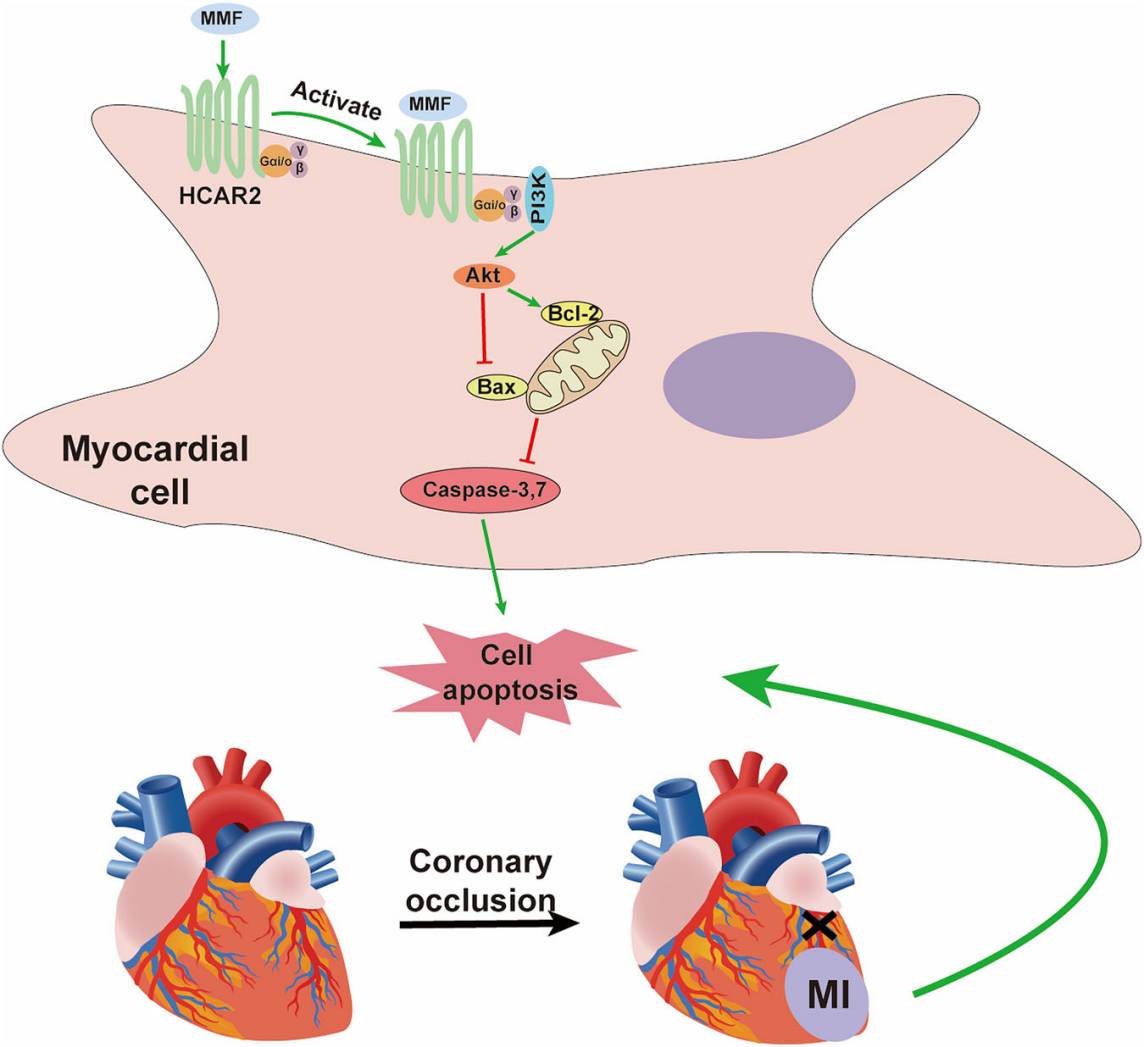
Proposed mechanism by which MMF protects the heart from post-MI injury. Schematic diagram illustrating the signaling pathway identified. Monomethyl fumarate (MMF) activates the HCAR2 (GPR109A) receptor on cardiomyocytes. HCAR2 is coupled to Gi proteins; upon MMF binding, the G αiβγ subunits stimulate the PI3K/Akt signaling cascade. Activated Akt phosphorylates and modulates downstream targets to promote cell survival—for example, Akt increases the level of anti-apoptotic Bcl-2 and inhibits pro-apoptotic factors like Bax and caspase-9. This leads to reduced activation of executioner caspases (casp-3, -7) and thus attenuates apoptosis of cardiomyocytes. By preventing ischemia-induced cardiomyocyte apoptosis, MMF limits infarct size and mitigates adverse left ventricular remodeling following an MI (depicted at bottom: coronary occlusion leads to MI and cell death, which MMF can ameliorate via the HCAR2-Akt pathway). This mechanism is supported by our experimental results and is consistent with known cardioprotective signaling pathways. MMF, monomethyl fumarate; MI, myocardial infarction; HCAR2, Hydroxycarboxylic Acid Receptor 2; Bax, Bcl-2–associated X protein; PI3K, Phosphatidylinositol 3-kinase.

Taken together, our data have proposed that MMF exerts cardioprotection after MI by engaging the HCAR2 receptor and activating PI3K/Akt signaling, which in turn inhibits apoptosis (Fig. 9). In this model, MMF binds to and activates HCAR2 on cardiomyocytes. HCAR2 is a Gi-protein-coupled receptor, and its activation releases Gβγ subunits that stimulate the phosphoinositide-3-kinase (PI3K) pathway, leading to phosphorylation of Akt [9, 10]. Active Akt then promotes cell survival through multiple mechanisms: it increases levels of the anti-apoptotic protein Bcl-2, and it phosphorylates/inactivates pro-apoptotic factors like Bax and caspase-9, thereby preventing the mitochondrial apoptotic cascade [10]. As shown in the diagram, the net effect is suppression of the executioner caspases (caspase-3 and -7) and a reduction in cardiomyocyte apoptosis. By rescuing cardiomyocytes from apoptotic death, MMF limits the loss of viable myocardium after coronary occlusion (MI), ultimately reducing infarct size and preventing the maladaptive remodeling that leads to heart failure [8, 10]. This multi-step mechanism is strongly supported by our experimental results: we observed that MMF maintained HCAR2 expression, enhanced Akt activity, shifted the Bax/Bcl-2 balance in favor of survival, and drastically reduced caspase activation and TUNEL-positive cell death in MI. Furthermore, blocking HCAR2 or Akt negated MMF’s beneficial effects, confirming the necessity of these components. MMF is known as an immunosuppressant that inhibits lymphocyte proliferation, but our study reveals a direct cardioprotective signaling role via HCAR2 in the heart. These insights suggest that repurposing MMF or targeting the HCAR2–Akt axis could be a promising strategy to protect the myocardium from ischemic injury and enhance cardiac recovery after MI. In summary, our study positions MMF as a compelling cardioprotective molecule that acts through a unique receptor-mediated mechanism. By activating HCAR2 and the PI3K/Akt pathway, MMF limits myocardial apoptosis and dysfunction following infarction. These insights bridge a mechanistic gap between MMF’s known biochemical actions and its therapeutic potential in heart disease. Given the extensive clinical experience with DMF in other diseases, there is a strong rationale to pursue MMF/DMF in preclinical large-animal models and eventually clinical trials in MI or cardiac surgery settings. If successful, this approach could inaugurate a novel class of cardioprotective therapy—one that repurposes an immunometabolic drug to combat ischemic injury in the heart.

## Conclusion

MMF markedly attenuates myocardial injury in a mouse MI model, preserving cardiac function and reducing cell death. The protective effect of MMF is mediated by the activation of HCAR2 (GPR109A) and its downstream PI3K/Akt signaling cascade, which promotes cardiomyocyte survival. This mechanism represents a previously unrecognized avenue for cardioprotection. Our findings suggest that MMF, the active metabolite of an existing anti-inflammatory drug, could be repurposed as a novel therapeutic agent to improve outcomes after MI. Further studies are warranted to validate these results in reperfusion models and to explore MMF’s efficacy when administered in a clinically relevant timeframe. If translated successfully, HCAR2 agonism might become a viable strategy to mitigate acute MI injury and enhance cardiac repair, complementing current reperfusion and pharmacological therapies.

## Materials and methods

### Animals and myocardial infarction model

All animal procedures were approved by the University of Calgary Animal Care Committee and conducted in accordance with the Canadian Council on Animal Care (CCAC) guidelines. Male C57BL/6 mice (8–10 weeks old) were used for the MI studies. Mice were anesthetized with isoflurane (1–2% inhalation in oxygen) and intubated for positive-pressure ventilation. A left thoracotomy was performed at the fourth intercostal space to expose the heart, and a permanent MI was induced by ligation of the left anterior descending (LAD) coronary artery. Specifically, a 7–0 polypropylene suture was placed around the LAD ~ 2 mm from the tip of the left atrium and tied securely to occlude coronary flow. Successful LAD ligation was confirmed by immediate blanching of the anterior left ventricular wall. Sham-operated mice underwent the same surgical procedure except that the suture was passed under the LAD without tying. Buprenorphine (0.1 mg/kg s.c.) was administered for postoperative analgesia, and mice were closely monitored during recovery.

Mice were randomly assigned to treatment with either MMF or vehicle. MMF (Sigma-Aldrich, St. Louis, MO, USA; 40 mg/kg in phosphate‑buffered saline (PBS)) or an equal volume of PBS (for controls) was administered intraperitoneally twice daily. In the MMF-treated group, dosing began 2 days prior to the MI surgery and continued for 5 days post-MI (for a total of 7 days of treatment). This dosing regimen (40 mg/kg i.p. BID) was chosen based on preliminary tolerance studies and is consistent with dosing used in previous cardioprotection investigations. All surgeries and drug administrations were performed in a randomized fashion, and investigators assessing cardiac function or tissue endpoints were blinded to the treatment groups. Ventricular remodeling at the experimental endpoint (day 5 post-MI) was assessed by gross morphological examination for left ventricular dilation, wall thinning, or cardiac rupture (Supplementary Fig. 1). Animals that died of rupture before terminal assessment were classified as remodeled.

### Echocardiographic assessment

Cardiac function was evaluated 5 days after MI by transthoracic echocardiography. Mice were lightly anesthetized with 1.0–1.5% isoflurane to maintain heart rate >450 beats/min and placed on a warmed platform in supine position. Echocardiography was performed using a Vevo 3000 high-frequency ultrasound imaging system (VisualSonics Inc., Toronto, ON, Canada) equipped with a 30 MHz transducer. Parasternal long-axis and short-axis B-mode images of the left ventricle (LV) were acquired, as well as M-mode recordings at the mid-ventricular level. Left ventricular end-diastolic volume (EDV) and ESV were measured from the long-axis images using the method of disks (modified Simpson’s rule) with the aid of Vevo LAB analysis software. Ejection fraction (EF) was calculated as (EDV − ESV)/EDV × 100%. LV anterior and posterior wall thicknesses at end-diastole and end-systole were measured from M-mode tracings at the mid-papillary level. All measurements were averaged over at least three consecutive cardiac cycles. Echocardiographic data analysis was performed by an experienced operator blinded to the experimental groups to ensure unbiased assessment.

### Hemodynamic pressure–volume loop analysis

Invasive hemodynamic measurements were conducted at the experimental endpoint (after echocardiography, typically 5–6 days post-MI) using pressure–volume (PV) loop analysis. Mice were anesthetized with isoflurane (2% in oxygen) and kept supine on a heating pad to maintain body temperature at 37 °C. A microtip conductance catheter (1.2 Fr Millar SPR-839, Millar Inc., Houston, TX, USA) was introduced into the right carotid artery and advanced retrogradely into the left ventricle. After stabilization, PV loops were recorded under steady-state conditions using the Millar MPVS Ultra system and LabChart Pro software. Heart rate, maximal and minimal left ventricular pressures, end-systolic and EDPs, stroke volume, cardiac output, and derived contractility indices (e.g., +dP/dt_max, –dP/dt_min) were obtained from the PV loops. Calibration of volume signals was performed by saline dilution and cuvette calibration according to the manufacturer’s protocol to allow calculation of absolute volumes. In some experiments, preload was transiently reduced (by gently compressing the inferior vena cava) to derive end-systolic pressure–volume relationships; however, the primary comparisons of contractile function were made from baseline PV loop parameters. All PV loop data were analyzed by an investigator blinded to the treatment groups. After completion of hemodynamic measurements, mice were euthanized by exsanguination under deep anesthesia, and hearts were rapidly excised for tissue analyses described below.

### HL-1 cardiomyocyte culture and hypoxia mimetic treatment

HL-1 cardiomyocytes (a mouse atrial cardiomyocyte cell line) were obtained from ATCC (Manassas, VA, USA) and cultured according to standard protocols. Cells were grown in Claycomb medium (Sigma-Aldrich, #51800 C) supplemented with 10% fetal bovine serum (FBS; Gibco, Waltham, MA, USA; #26140), 2 mM L-glutamine (Gibco, Waltham, MA, USA; #25030081), 100 U/mL penicillin and 100 μg/mL streptomycin (Gibco, Waltham, MA, USA; #15140122), and 0.1 mM norepinephrine (Sigma-Aldrich, #A7257). Culture flasks were pre-coated with 0.02% gelatin and 5 μg/mL fibronectin (Sigma-Aldrich, #F1141) to promote HL-1 cell adhesion. Cells were maintained at 37 °C in a humidified 5% CO₂ atmosphere and passaged at ~80–90% confluency. All HL-1 cells used in experiments were from low passage numbers (<P20), were confirmed mycoplasma-negative (MycoAlert kit, Lonza), and were authenticated by the provider.

To simulate ischemic stress in vitro, a chemical hypoxia model was used. Confluent HL-1 cells were exposed to cobalt(II) chloride (CoCl₂; 200 μM, Sigma-Aldrich, #255599) for 12 h to mimic hypoxia by stabilizing HIF-1α. Experimental groups were established to evaluate the effects of MMF and pathway inhibitors as follows: (1) Normoxia control: Cells maintained in normoxic conditions (no CoCl₂, no treatment). (2) CoCl₂ alone (Hypoxia): Cells exposed to CoCl₂ (200 μM, 12 h) without additional treatment. (3) CoCl₂ + MMF: Cells exposed to CoCl₂ with MMF treatment (50 μM, Sigma-Aldrich, # 651419) added 1 h before CoCl₂ and maintained during the 12 h exposure. (4) CoCl₂ + MMF + PTX: Cells pre-treated with pertussis toxin (PTX, 100 ng/mL, Cayman Chemical, #19546) 2 h prior to MMF to inhibit Gi/o protein-coupled receptor signaling, then treated with MMF (50 μM) 1 h before and during CoCl₂ exposure. (5) CoCl₂ + MMF + LY294002: Cells pre-treated with the PI3K inhibitor LY294002 (10 μM, Sigma-Aldrich, # 124011) 1 h prior to MMF, then treated with MMF and CoCl₂ as above (to block Akt signaling). (6) CoCl₂ + MMF + HCAR2 siRNA: Cells transfected with a small interfering RNA targeting HCAR2 (Thermo Fisher, #1320001) 48 h before treatment to knock down HCAR2 expression. After confirming knockdown, cells were treated with MMF and CoCl₂ as in group 3. (7) CoCl₂ + Niacin: Cells treated with niacin (nicotinic acid, 1 mM, Sigma-Aldrich, #72309) added 1 h before and during CoCl₂ exposure, as a positive control for HCAR2 receptor activation (niacin is a known HCAR2 agonist).

Throughout all cell experiments, MMF (50 μM) was obtained from Sigma-Aldrich (# 651419) and prepared fresh in culture medium. PTX and LY294002 stock solutions were prepared according to the manufacturers’ instructions (PTX reconstituted in water; LY294002 in DMSO) and added such that the final DMSO vehicle concentration did not exceed 0.1%. Control groups received equivalent vehicle for each reagent. After the 12 h CoCl₂ exposure, cells were immediately processed for viability and apoptosis assays or lysed for molecular analyses as described below.

### Cell viability and apoptosis assays

Cell injury and death in HL-1 cells were quantified by multiple complementary assays. Apoptosis was assessed using terminal deoxynucleotidyl transferase dUTP nick-end labeling (TUNEL). Treated cells on chamber slides were fixed with 4% paraformaldehyde and processed with a TUNEL staining kit (Roche Diagnostics, Indianapolis, IN, USA; catalog #12156792910) according to the manufacturer’s instructions. Fluorescein-labeled dUTP was incorporated at DNA strand breaks, and nuclei were counterstained with 4′,6-diamidino-2-phenylindole (DAPI). Slides were then analyzed by laser scanning cytometry to quantify the percentage of TUNEL-positive nuclei. Specifically, stained slides were scanned using a laser scanning cytometer (LSC, Thorlabs CompuCyte) that detects DAPI (total nuclei) and fluorescein (TUNEL) signals across the entire slide, as we previously described [15, 16]. The fraction of apoptotic cells was calculated as TUNEL-positive nuclei divided by total nuclei, and at least 5 fields per sample were analyzed to obtain a representative value.

Cell viability was evaluated by the methyl-thiazolyl tetrazolium (MTT) assay. After treatments, MTT reagent (0.5 mg/mL final concentration, Sigma-Aldrich, #T8877) was added to cells and incubated for 4 h at 37 °C. Living cells reduce MTT to a purple formazan product. The reaction was stopped by removing the medium and dissolving the formazan crystals in DMSO (150 μL/well). The absorbance of each well was measured at 570 nm (reference 630 nm) using a microplate spectrophotometer, and values were normalized to the normoxia control group (set as 100% viability).

### Western blot analysis

Protein expression of signaling and apoptosis-related proteins was examined by Western blotting both in heart tissues and HL-1 cells. Mouse heart tissue from the infarct and border zone region (approximately the anterior LV wall) was dissected 5 days post-MI (immediately after PV loop measurements) and snap-frozen in liquid nitrogen. Tissue samples and cultured HL-1 cells (harvested after treatments) were homogenized in ice-cold RIPA lysis buffer (50 mM Tris-HCl pH 7.4, 150 mM NaCl, 1% NP-40, 0.5% sodium deoxycholate, 0.1% SDS) supplemented with protease and phosphatase inhibitor cocktails (Roche, # 4906845001). Lysates were incubated on ice for 30 min and cleared by centrifugation (14,000 × *g*, 15 min, 4 °C). The supernatant protein concentration was determined by a bicinchoninic acid (BCA) assay (Pierce, Thermo, #A55864). Equal amounts of protein (30 μg per sample) were mixed with Laemmli sample buffer, boiled, and separated by SDS-PAGE on 4–12% polyacrylamide gels. Proteins were then transferred onto PVDF membranes (Millipore, Burlington, MA, USA) using a wet transfer apparatus.

Membranes were blocked with 5% non-fat milk in TBS-T buffer for 1 h at room temperature, then incubated overnight at 4 °C with primary antibodies against the proteins of interest. The following primary antibodies were used: HCAR2 (hydroxycarboxylic acid receptor 2, rabbit polyclonal, 1:1000, Invitrogen, Carlsbad, CA, USA; cat. # PA5-90579), phospho-Akt (Ser473) (rabbit monoclonal, 1:1000, Cell Signaling Technology; cat. #4060), total Akt (rabbit monoclonal, 1:1000, Cell Signaling Technology; #9272), Bcl-2 (mouse polyclonal, 1:200, Santa Cruz Biotechnology; #sc-7382), Bax rabbit monoclonal, (1:1000, Cell Signaling Technology; #2772), caspase-3 (full-length, rabbit polyclonal, 1:1000, Cell Signaling Technology; #2772), cleaved caspase-3 (Asp175 fragment, rabbit monoclonal, 1:1000, Cell Signaling Technology; #9661), and GAPDH (glyceraldehyde-3-phosphate dehydrogenase, mouse monoclonal, 1:1000, Cell Signaling Technology; #2118). After primary antibody incubation, membranes were washed and then probed with appropriate horseradish peroxidase (HRP)-conjugated secondary antibodies (goat anti-rabbit IgG-HRP or goat anti-mouse IgG-HRP, Invitrogen; 1:5000, cat. #65-6120 and 62-6520) for 1 h at room temperature. Immunoreactive bands were visualized using an enhanced chemiluminescence substrate (ECL Plus, Thermo Fisher) and captured on a ChemiDoc MP imaging system (Bio-Rad).

To quantify protein levels, band intensities on Western blots were analyzed by densitometry using ImageJ software (National Institutes of Health, Bethesda, MD, USA). Each target protein band intensity was normalized to its corresponding GAPDH band to control for loading differences. Data from at least 3 independent experiments or biological replicates were averaged for statistical analysis.

### Immunofluorescence and confocal microscopy

Immunofluorescence staining was performed to localize HCAR2 and β-catenin in heart tissue sections from the infarct area. After hemodynamic assessment, hearts were arrested in diastole with KCl, excised, and fixed in 10% neutral-buffered formalin for 24 h. Tissues were then processed and embedded in paraffin; 5 μm thick transverse sections from the infarct border zone were mounted on charged slides. For antigen retrieval, deparaffinized sections were heated in citrate buffer (10 mM sodium citrate, pH 6.0) at 95 °C for 15 min, then cooled and rinsed in PBS. Sections were permeabilized with 0.2% Triton X-100 for 10 min and blocked with 5% normal goat serum (Vector Laboratories) for 1 h at room temperature to prevent nonspecific binding. Slides were then incubated overnight at 4 °C with primary antibodies against HCAR2 (rabbit polyclonal, 1:200, Invitrogen; # PA5-90579) and β-catenin (mouse monoclonal, 1:200, Cell Signaling Technology; #37447). The next day, sections were washed and incubated with species-appropriate secondary antibodies conjugated to fluorophores (Rhodamine Red anti-rabbit IgG, Invitrogen, 1:500, #R6394 and Alexa Fluor 488 anti-mouse IgG, Cell Signaling Technology; 1:500, #4408) for 1 h in the dark. Nuclei were counterstained with DAPI (4′,6-diamidino-2-phenylindole, 1 μg/mL) for 5 min. After final washes, slides were coverslipped with antifade mounting medium.

Fluorescent images were acquired using a confocal laser-scanning microscope (Zeiss LSM880, Carl Zeiss, Germany; or equivalent) equipped with 405 nm, 488 nm, and 561 nm lasers. Images of the infarct region were captured with a 40× oil-immersion objective, and settings for laser power, detector gain, and pinhole were kept constant between groups. For each heart, multiple fields in the infarct border zone were imaged. Co-localization and cellular localization of HCAR2 (red signal) and β-catenin (green signal) were assessed qualitatively. All image acquisition and analyses were performed by an observer blinded to the treatment groups. Negative control sections (omission of primary antibodies) showed no significant fluorescence, confirming antibody specificity.

### Statistical analysis

Data are expressed as mean ± standard error of the mean (SEM). Statistical analyses were performed using GraphPad Prism 9.0 (GraphPad Software, San Diego, CA, USA). For multiple-group comparisons, one-way analysis of variance (ANOVA) was used followed by Tukey’s post hoc test for between-group differences. For comparisons between two groups, an unpaired two-tailed Student’s *t*-test was applied. If data did not meet normality or equal variance assumptions, a non-parametric Kruskal–Wallis test was employed (with Dunn’s post hoc test for multiple comparisons). A *p* < 0.05 was considered statistically significant for all analyses. Each experiment was repeated at least three times or included multiple biological replicates as indicated, to ensure reproducibility and robust statistical power.

## Supplementary information

Supplemental Figure

Original Data

## Data Availability

The data used to support the findings of this study are available from the corresponding author upon request.
