# Supplementary material for: Monomethyl fumarate confers cardioprotection after myocardial infarction via HCAR2-dependent activation of PI3K/Akt signaling
Source: Cell Death Discov. 2025 Dec 30;12:63. doi: 10.1038/s41420-025-02927-6 (PMC12847698; doi:10.1038/s41420-025-02927-6)
Supplement: Supplementary file 1 — Supplemental Figure [file 41420_2025_2927_MOESM1_ESM.docx]

**
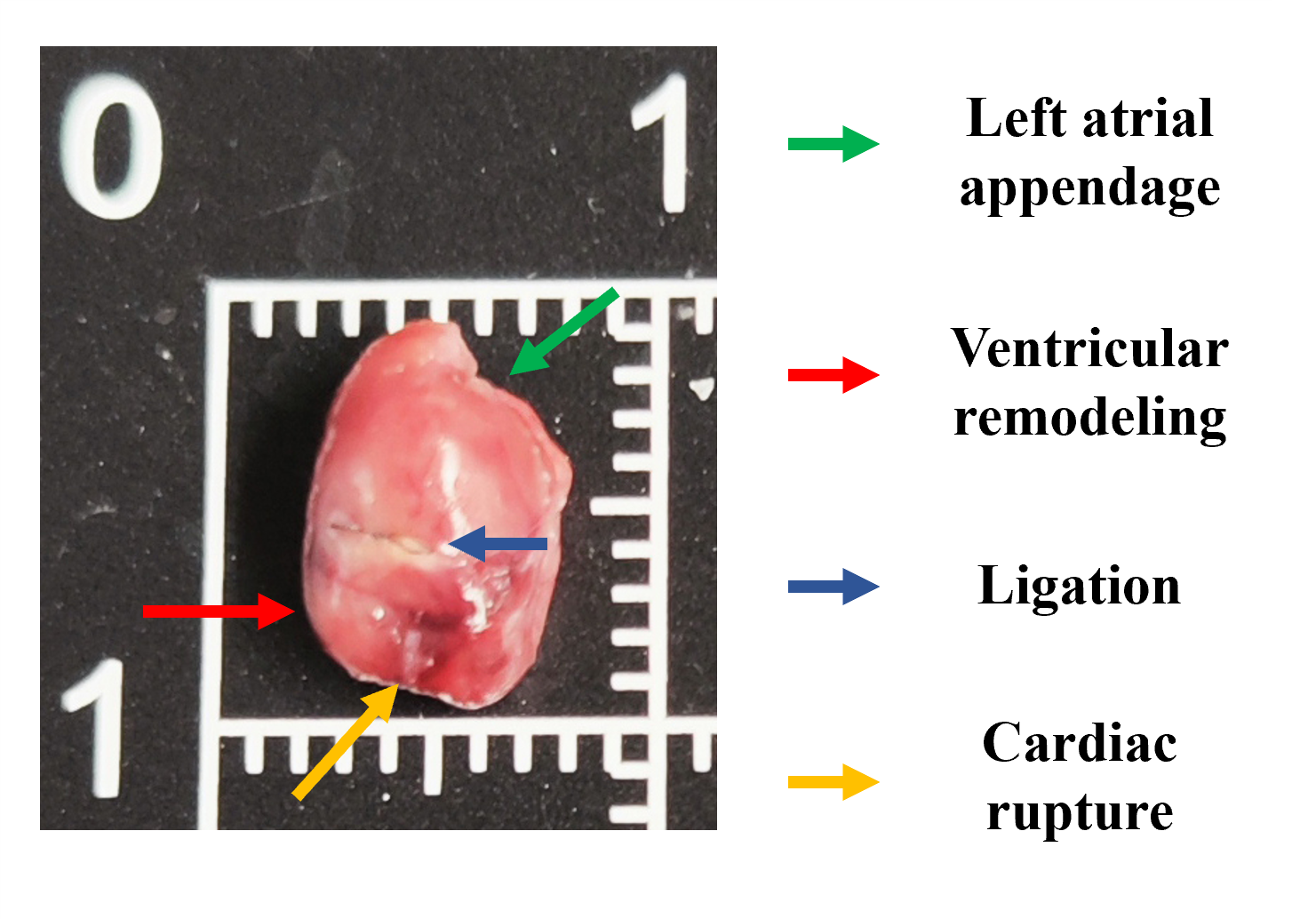
**

**Supplementary Figure S1. Representative gross anatomy of hearts in MI group**

In the MI group, one mouse died on postoperative day 2 and another on day 4. Cardiac rupture with evident ventricular remodeling was observed in the hearts of these deceased mice; therefore, these samples were included in the remodeling statistics. In the figure, green arrows indicate the left atrial appendage, which serves as an anatomical landmark. Blue arrows indicate the ligation site of the LAD coronary artery. Red arrows highlight morphological changes associated with ventricular remodeling. Yellow arrows point to the site of cardiac rupture.
