# Supplementary material for: Monomethyl fumarate confers cardioprotection after myocardial infarction via HCAR2-dependent activation of PI3K/Akt signaling
Source: Cell Death Discov. 2025 Dec 30;12:63. doi: 10.1038/s41420-025-02927-6 (PMC12847698; doi:10.1038/s41420-025-02927-6)

Figure 3C

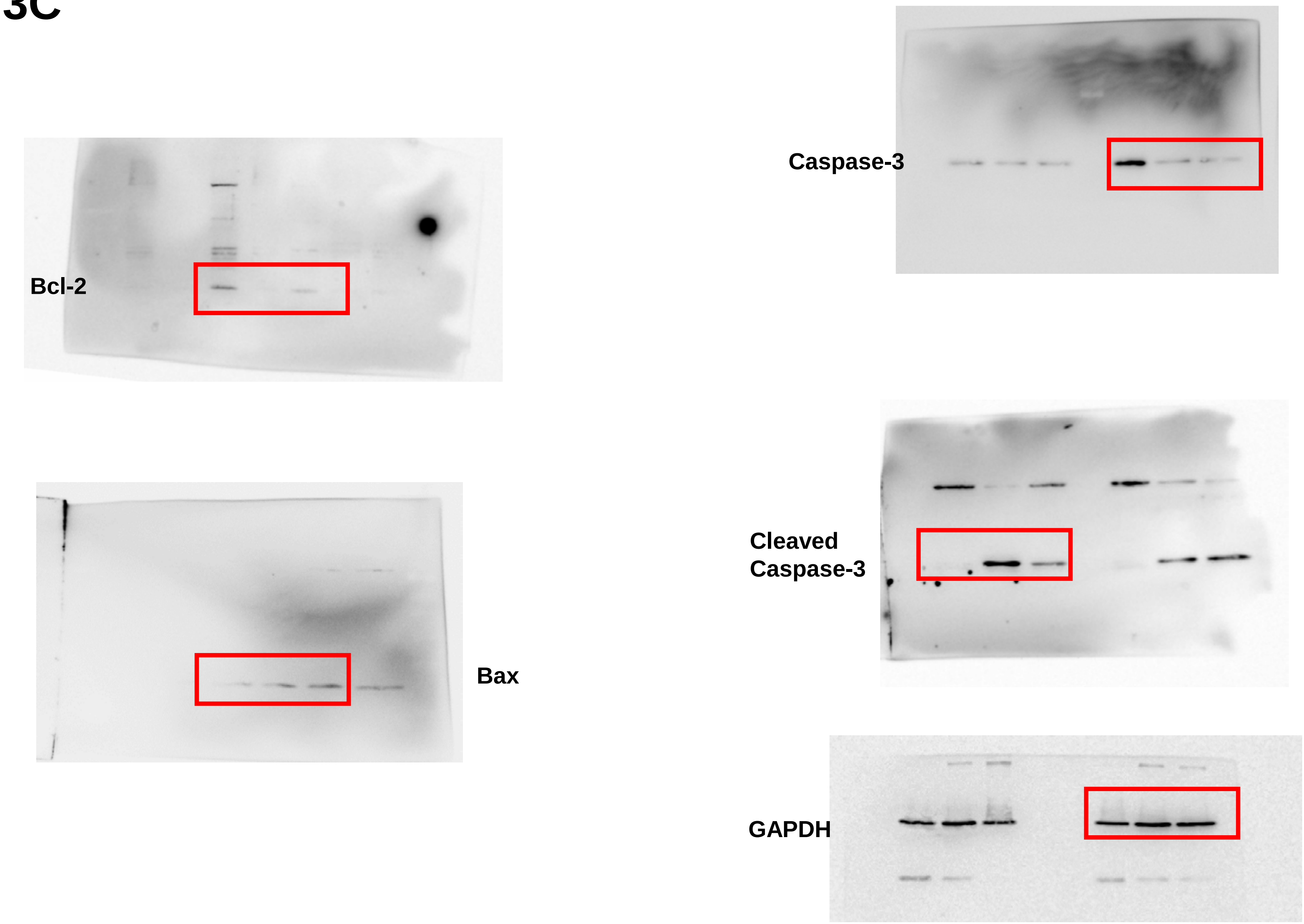

Figure 4D

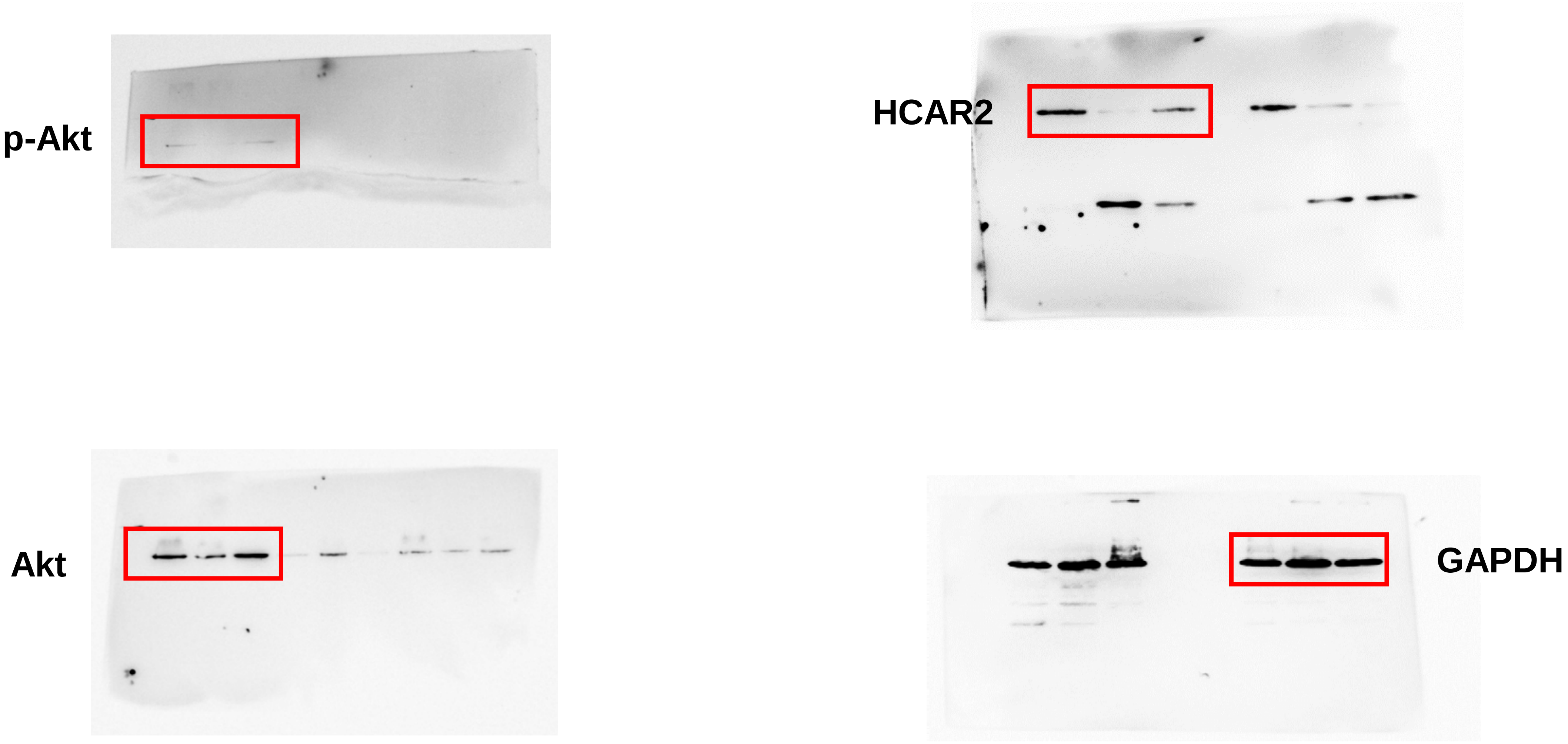

Figure 5B

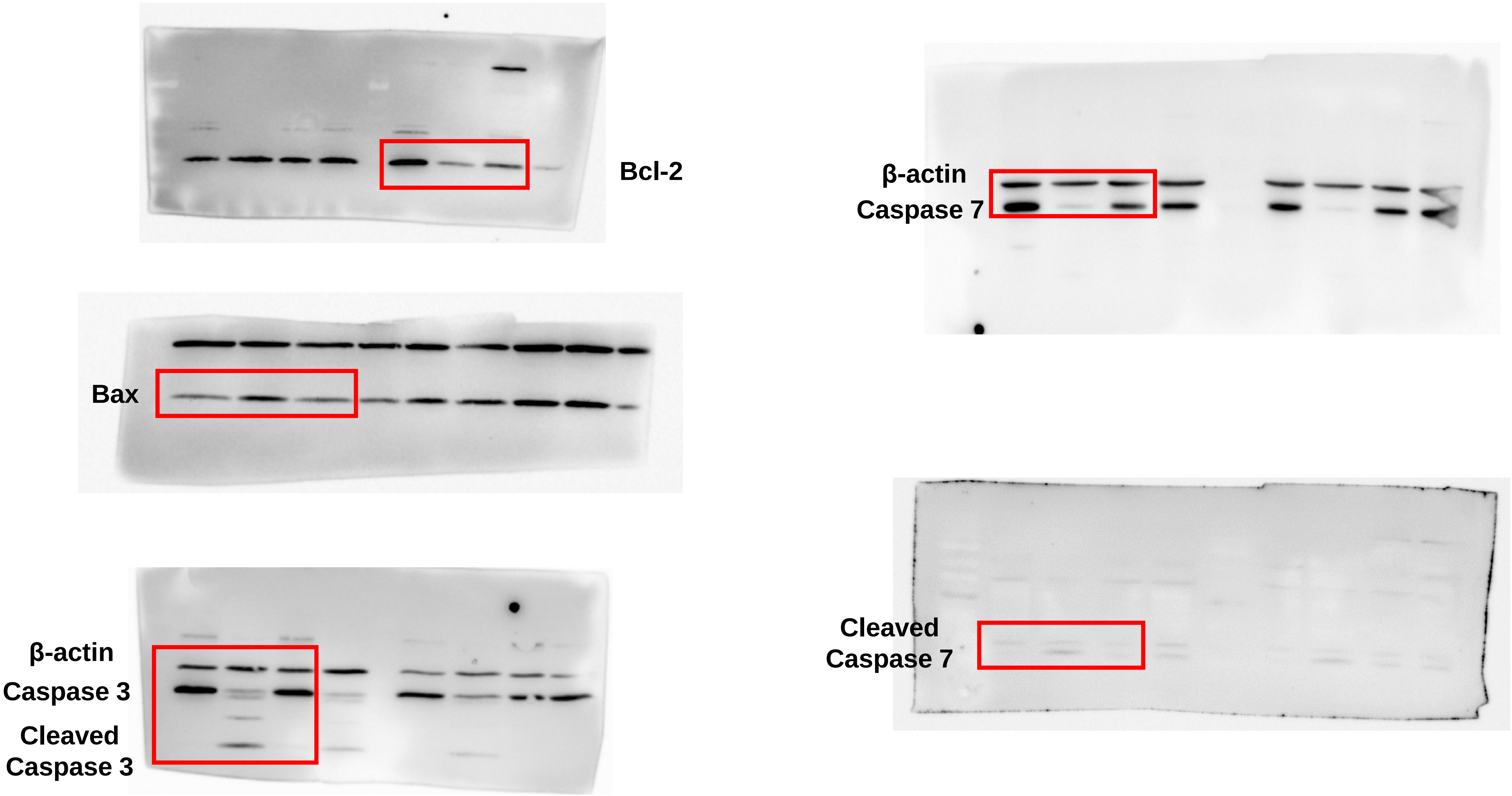
Bcl-2
Bax
β-actin
Caspase 3
Cleaved
Caspase 3
β-actin
Caspase 7
Cleaved
Caspase 7

Figure 6F

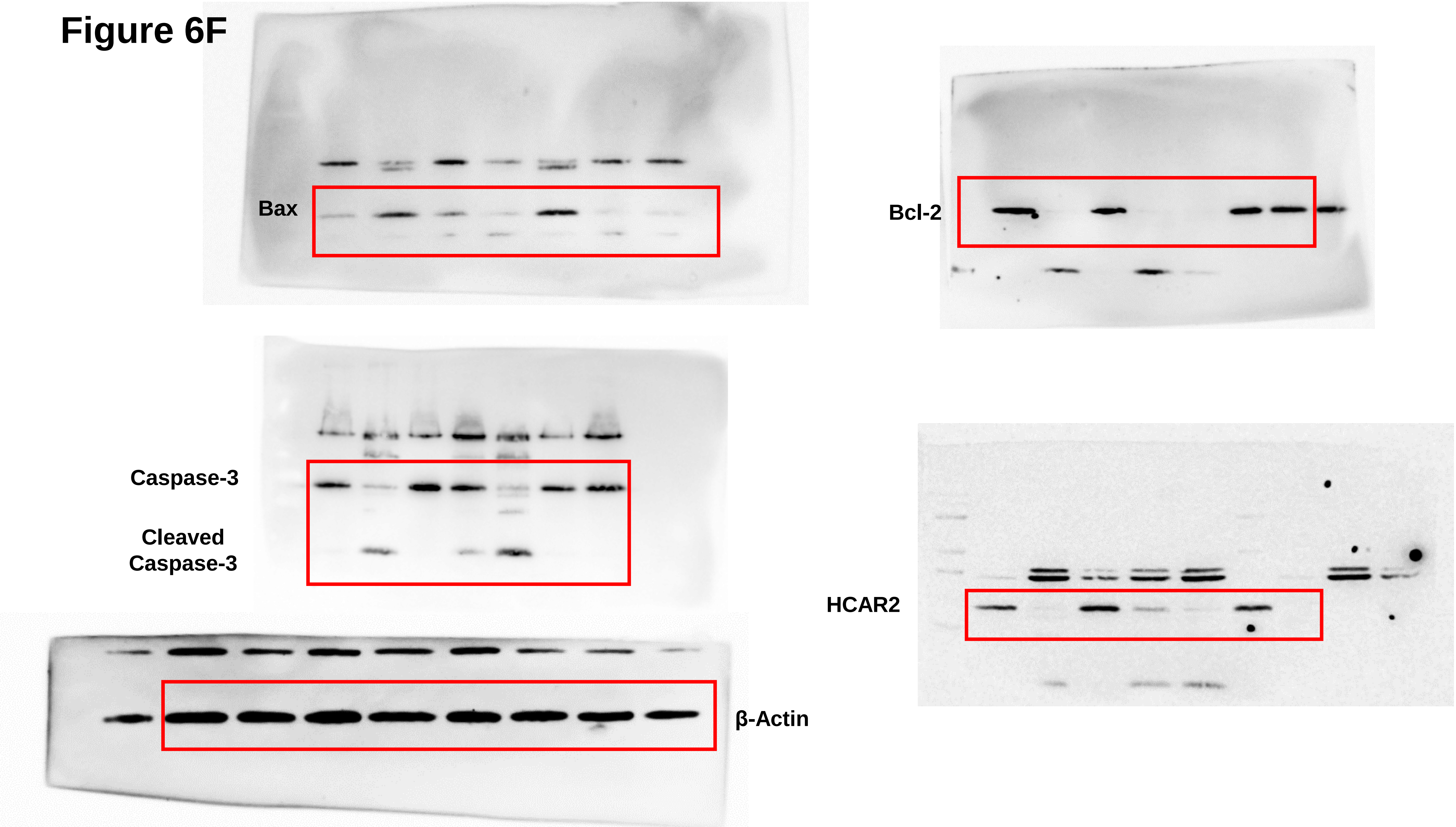

Figure 6D

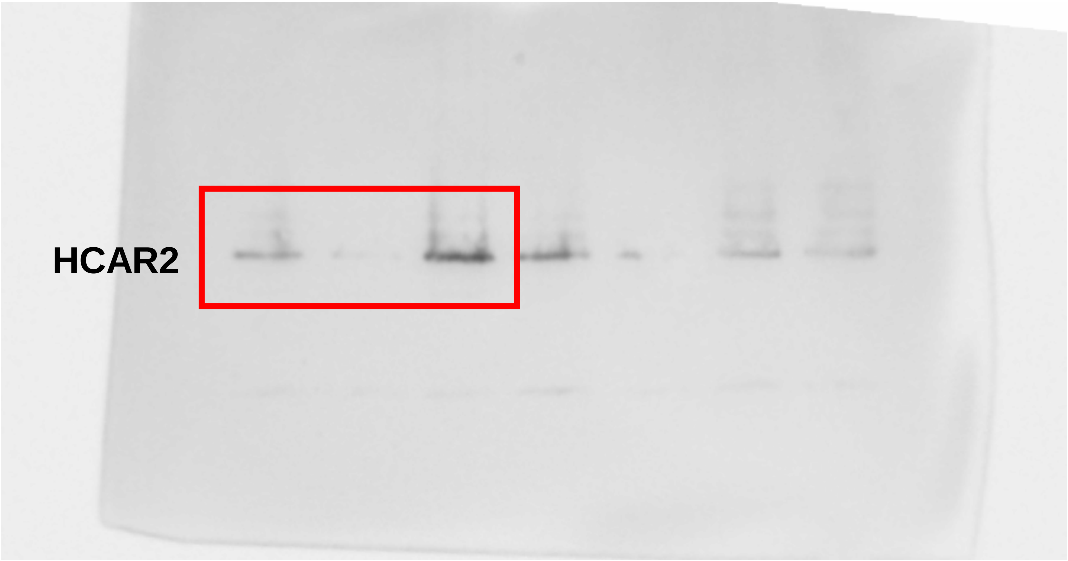

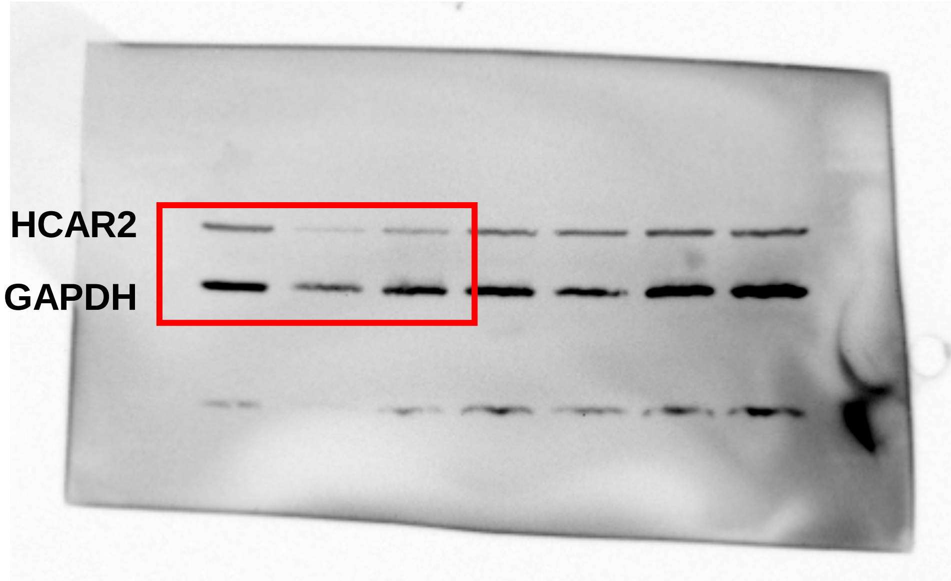

Figure 8A

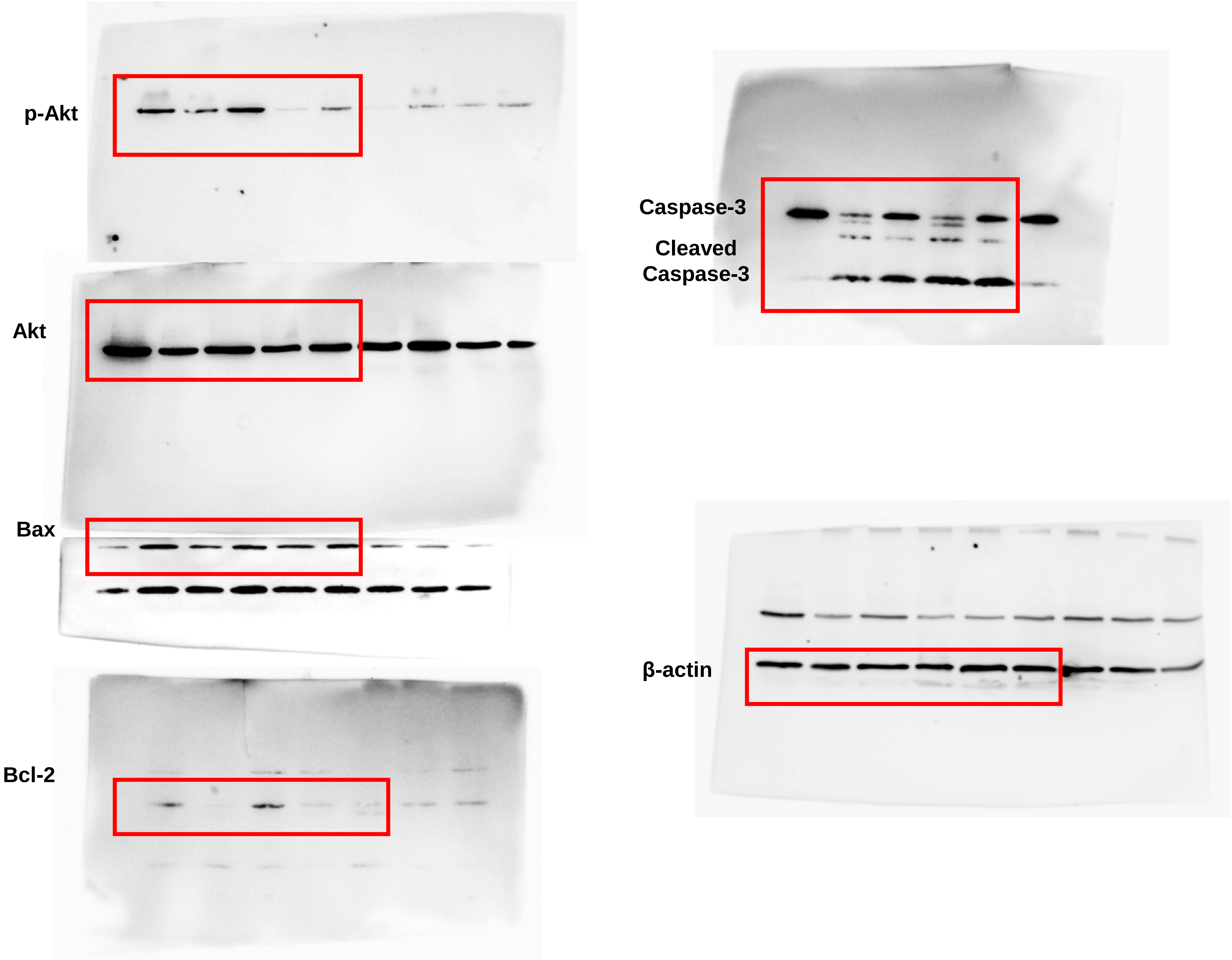

Supplement: Supplementary file 2 — Original Data [file 41420_2025_2927_MOESM2_ESM.pdf]
